# Selective Targeting of the TPX2 Site of Importin-α Using Fragment-Based Ligand Design

**DOI:** 10.1002/cmdc.201500014

**Published:** 2015-04-20

**Authors:** Rhian S Holvey, Eugene Valkov, David Neal, Murray Stewart, Chris Abell

**Affiliations:** [a] Department of Chemistry, University of CambridgeLensfield Road, Cambridge, CB2 1EW (UK); [b] MRC Laboratory of Molecular BiologyFrancis Crick Avenue, Cambridge, CB2 0QH (UK); [c] University of CambridgeDepartment of Oncology, Box 279, Addenbrooke's Hospital, Hills Road, Cambridge, CB2 0QQ (UK)

**Keywords:** cancer, fragment-based ligand design, nuclear transporters, protein–protein interactions, structure-guided ligand design

## Abstract

Protein–protein interactions are difficult therapeutic targets, and inhibiting pathologically relevant interactions without disrupting other essential ones presents an additional challenge. Herein we report how this might be achieved for the potential anticancer target, the TPX2–importin-α interaction. Importin-α is a nuclear transport protein that regulates the spindle assembly protein TPX2. It has two binding sites—major and minor—to which partners bind. Most nuclear transport cargoes use the major site, whereas TPX2 binds principally to the minor site. Fragment-based approaches were used to identify small molecules that bind importin-α, and crystallographic studies identified a lead series that was observed to bind specifically to the minor site, representing the first ligands specific for this site. Structure-guided synthesis informed the elaboration of these fragments to explore the source of ligand selectivity between the minor and major sites. These ligands are starting points for the development of inhibitors of this protein–protein interaction.

## Introduction

Many biological processes rely on the formation of protein–protein complexes. These interactions are potential therapeutic targets, but their diversity presents a significant challenge to the development of small-molecule inhibitors.[1] These challenges are mainly due to proteins being relatively large and having interaction surfaces that are, relative to enzyme active sites, frequently rather featureless, and which often rely on interactions involving noncontiguous amino acids. Moreover, in contrast to the case for many enzymes and protein transmembrane channels, activity-based assays cannot usually be used to identify inhibitors.[2] Despite these challenges, progress is being made in targeting protein–protein interactions (PPIs).[3] One of the methods showing significant promise is fragment-based ligand discovery, in which binding fragments (*M*_r_<300 Da) are identified through biophysical screens and elaborated into more potent molecules using structure-based ligand design.[4]

In addition to the general challenges of PPIs, there is also the potential issue of site selectivity. Many target proteins interact with a number of different partners and may possess multiple interaction sites. These problems of promiscuous binding and multiple sites increase the risk of inhibitors also disrupting beneficial interactions.[5] The nuclear transporter karyopherin-α/importin-α, which is of increasing interest as an anticancer and antiviral drug target,[6] exemplifies many of these problems. In addition to having an essential role in mediating the import of cargo proteins into the nucleus by binding to nuclear localisation signals (NLSs) that are generally specific clusters of positively charged amino acids,[7] importin-α also regulates the function of the non-motor spindle assembly factor TPX2 (**t**arget **p**rotein for ***X**enopus* kinesin-like protein **2**). Importin-α is a banana-shaped protein that is constructed from a series of 10 repeating structural motifs called *armadillo repeats*; it binds both the NLSs of nuclear import cargo proteins and TPX2 using sites on its inner concave surface. All NLSs bind to the major binding site, spanning armadillo repeats 1–4, and some also have a small additional contribution from the minor site, spanning repeats 6–8.[8] TPX2, however, binds primarily to the minor site of importin-α, thereby offering an opportunity for selectivity.

Both the major and minor sites on importin-α interact with unstructured peptides on either NLSs or TPX2, which is in contrast to many other PPIs in which both partners are structured, as observed, for example, with p53 or Bcl-x_L_, in which the binding motif is based on an α-helix.[9] Interactions based on such unstructured peptides are more challenging to target, but some progress has been described for the BRCA2–RAD51 interaction, for example.[10]

**Figure 1:**
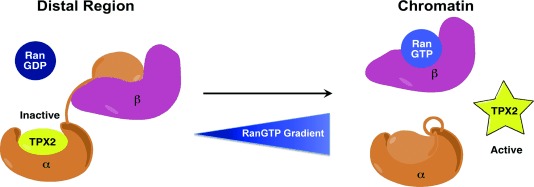
Schematic of the role of importin-α and RanGTP in the regulation of TPX2. In mitosis, after the nuclear membrane has been disassembled, RanGTP is maintained at high concentration in the vicinity of chromatin. In distal regions, Ran is predominantly in the GDP-bound form, leading to a gradient of RanGTP concentration. RanGDP does not bind importin-β, allowing it to bind the autoinhibitory IBB domain of importin-α that exposes the NLS binding sites, and so allows importin-α to sequester and inhibit TPX2 activity. This ternary complex remains stable until it diffuses into the high concentration of RanGTP near the chromosomes, where RanGTP binds to importin-β, releasing the IBB domain and displacing TPX2, which thereby becomes active, allowing for site-specific spindle assembly.

TPX2 is essential for the formation of an ordered spindle and successful mitosis,[11] and its regulation by importin-α is the major component of RanGTP-mediated spindle assembly (Figure 1), a process on which cancer cells rely more heavily than other cells.[12] As the transport and mitotic roles of importin-α employ two different sites on the protein, the TPX2–importin-α interaction has potential as an anticancer target, provided selectivity for the minor site over the major site can be achieved. Previous work had focused on establishing the consensus peptide sequence KRXF/Y/W[13] for recognition of the minor site over the major site,[14] but no small molecules have yet been designed specifically for this site. Herein we present a fragment-based approach to the development of the first site-specific ligands of the minor site TPX2–importin-α interaction and provide validation for this approach to targeting complex proteins such as importin-α.

## Results and Discussion

### Identification of importin-α binding fragments by ligand- observed NMR screening of a thermal-shift-enriched library

Previous reports have shown that the hit rate for PPIs is typically lower than for enzyme targets, leading to the development of modified versions of fragment-screening cascades to minimise the amount of false negatives and positives.[10] For TPX2–importin-α, a 1248-member ‘rule-of-three’-compliant[15] fragment library was screened using fluorescence-based thermal shift[16] to generate an enriched library of 140 compounds. To prevent the autoinhibition that is crucial for expelling NLSs in the nucleus, an importin-α construct (Δ*IBB*-importin-α) was used from which the importin-β binding domain (IBB) had been excised.[14] Compounds were excluded from the enriched library if they were shown to denature or aggregate the protein at room temperature, or otherwise give strong negative thermal shifts, as these may otherwise have generated false positives in subsequent ligand-observed NMR experiments. The enriched 140-fragment library was screened by the orthogonal ligand-observed NMR techniques saturation transfer difference (STD),[17] water–ligand observed via gradient spectroscopy (WaterLOGSY)[18] and Carr–Purcell–Meiboom–Gill (CPMG) spin-echo train sequence.[19] From the 140 compounds, 78 showed binding to importin-α, of which 35 showed displacement by a 20-residue minor site binding TPX2 peptide (*K*_d_=350 nm) in at least one technique, suggesting that these fragments were binding the TPX2 binding site on importin-α.

As an early study of the structure–activity relationship, 15 commercial compounds with scaffold similarity to **1** (as the fragment showing the largest changes in binding and displacement signals across all three ligand-observed NMR techniques; see Supporting Information Figure S1) were screened, identifying a further nine compounds with good binding and displacement, giving a total of 44 fragments (structures listed in Supporting Information Table S1). The 44 potential importin-α minor site binding fragments identified in these screens were then ranked by competitive isothermal titration calorimetry (ITC).[20] As both thermal shift and NMR experiments were performed at pH 8.0, competitive ITCs were also conducted at this pH; however, all but two fragments (**6** and **10**) showed only very weak (>15 mm competitive *K*_d_) or no competition with the TPX2 minor site binding peptide (Supporting Information Table S2). To prioritise compounds for crystallography, competitive ITC experiments were then performed at pH 6.0, the pH to be used for the crystallographic soaking trials, removing those that were not soluble to 10 mm in the ITC buffer. From these studies 10 fragments (compounds **1**–**10**) were shown to have *K*_d_≤10 mm at pH 6.0 (Table 1) and were prioritised for structural studies.

**Table 1:** Structures and *K*_d_ values of fragments 1–10 that were subsequently prioritised for crystallography.

Compd	Structure	*K*_d_ [mm][a]
**1**	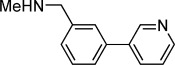	8.7±0.8
**2**	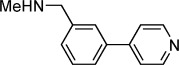	6.0±0.6
**3**	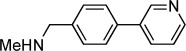	9.2±0.7
**4**	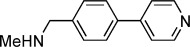	10.0±1.2
**5**	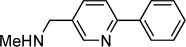	9.1±0.8
**6**	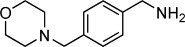	8.2±0.7
**7**	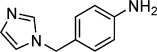	3.2±0.3
**8**	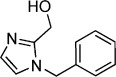	9.7±0.9
**9**	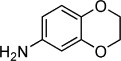	4.7±0.4
**10**	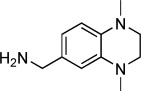	5.2±0.5

[a] Determined by competitive ITC at pH 6.0; error values reported are compound errors from the standard deviations of two replicate control experiments and two replicate competitive fragment experiments.

### Structure of compound 1 shows selective binding to the minor site through Glu396

**Figure 2:**
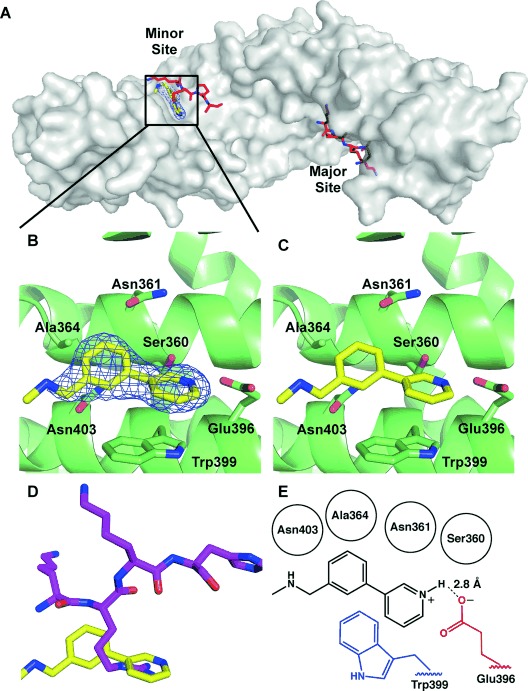
Structural validation of fragment hit 1. A) Compound 1 (yellow) bound specifically to the minor site of importin-α (grey surface). 2*F*_o_−*F*_c_ density for compound 1 is shown at 1.0 *σ* (blue mesh). Red sticks represent the minor and major site binding portions of the cargo protein nucleoplasmin from the PDB structure 1EJY.[30] B) Fragment 1 (yellow) with the 2*F*_o_−*F*_c_ density shown contoured around the ligand at 1.0 *σ* (blue mesh) bound to the importin-α minor site (green ribbon and sticks). C) Fragment 1 bound to the importin-α minor site without electron density. D) Fragment 1 structure overlaid with the key TPX2 residues (magenta) from the TPX2–importin-α crystal structure 3KND.[14] E) Schematic to show the key interactions with fragment 1: red residues make hydrogen bonding or salt-bridge contacts, blue residues are involved in π–π stacking interactions, and residues denoted by black circles form nonpolar interactions with the ligand.

Fragments **1**–**10** were individually soaked into unliganded crystals of importin-α. X-ray datasets of resolution between 2.2 and 2.5 Å were collected for all except for fragments **9** and **10**. Inspection of the electron density maps only showed clear difference electron density for fragment **1**, which was refined to 2.4 Å resolution. Attempts to co-crystallise the remaining fragments under a variety of conditions were unsuccessful (Supporting Information). Significantly, the co-crystal structure of importin-α with fragment **1** revealed difference density for the compound only in the minor site of importin-α with no significant density peaks in the major site, suggesting that the initial fragment demonstrated considerable site selectivity (Figure 2 A).

Fragment **1** binds in the key minor site ‘hot spot’ positioned such that the pyridine nitrogen atom is within hydrogen bonding or salt-bridge distance (2.8 Å) of the carboxylate group of the defining minor site residue Glu396 (where the crucial arginine of TPX2 also forms interactions; Figure 2 B–D). The aromatic rings of the fragment form a π–π stacking interaction with Trp399, with the pyridine ring overlapping the indole nitrogen ring at a distance of 3.3 Å and an angle of 101°, and the phenyl ring overlaying edges with the indole phenyl ring at a distance of 3.3 Å and an angle of 143°. The π–π stacking interaction between both rings of the fragment and the Trp399 indole results in a twist between the fragment aromatic rings with a torsion angle of 38° (Figure 2 E).

### Fragment merging with TPX2 lysine

**Figure 3:**
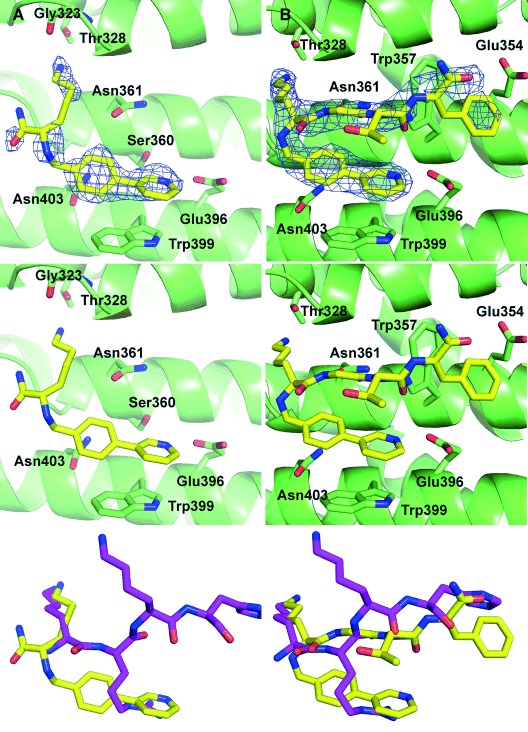
Structures of compounds A) 11 and B) 12 bound to the minor site of importin-α. Top: ligand (yellow) with the 2*F*_o_−*F*_c_ density shown contoured around the ligand at 1.0 *σ* (blue mesh) bound to the importin-α minor site (green ribbon and sticks). Middle: ligand bound in minor site without density. Bottom: ligand structure overlaid with the key TPX2 residues (magenta) from the TPX2–importin-α crystal structure 3 KND.[14]

After studying the overlay of fragment **1** on the previously reported TPX2–importin-α structure,[14] compound **11** was synthesised by merging the fragment with the lysine of the key tetrapeptide KRXF/Y/W consensus sequence for TPX2 via an amine linker using a reductive amination approach. Direct ITC at pH 6.0 gave a *K*_d_ value of 4.0 mm for **11**, a modest increase on the *K*_d_ value of 8.7 mm for fragment **1**. To understand how **11** was binding, a co-crystal structure of it bound to importin-α (Figure 3 A) was determined to 2.5 Å. This showed that **11** maintained the key pyridine Glu396 interaction as for compound **1** (2.8 Å) as well as maintaining the π–π stacking interaction with Trp399 (the pyridine ring again overlapping the indole nitrogen ring at a distance of 3.5 Å and an angle of 96° and the phenyl ring overlaying edges with the indole phenyl ring at a distance of 3.3 Å and an angle of 118°). These changes result in a good overlay of the fragment portion of **11** with the structure of **1** (Supporting Information Figure S2); once again the torsion angle between the two aromatic rings was 40° similar to fragment **1** (38°), suggesting the lysine was able to reach the new pocket without changing the fragment orientation. This suggests that the fragment, despite its low affinity, is a good anchor in this key hotspot. As for the structure of **1**, there was no density in the importin-α major site for **11**, suggesting that the new ligand maintains specificity to the minor site. The structure of **11** showed reasonable density for the amine of the lysine group, indicating that it is reaching the desired pocket and forming a hydrogen bond with the carbonyl group of Gly323 (3.0 Å). Despite these additional interactions, the increase in affinity of **11** for importin-α was modest, possibly due to the alternative orientation of the lysine backbone (∼160° flip) relative to the native TPX2 peptide (Figure 3 A).

### Extending the peptide chain

To assess whether the orientation of the lysine backbone in **11** was important, and to probe other interactions in the minor site, a longer merged peptide of the form fragment-KGTF **12** (with glycine replacing the arginine, the interaction of which is mimicked by the N-terminal fragment and the TF from human TPX2) was synthesised and its binding analysed. Direct ITC of **12** at pH 6.0 gave a *K*_d_ value of 3.3 mm, showing no improvement on **11**. The co-crystal structure of **12** bound to importin-α was determined to 2.3 Å resolution (Figure 3 B). Analysis of the ligand conformation revealed that the extended peptide had caused the lysine backbone to undergo a ∼150° flip to orient with the native TPX2 tetrapeptide, while the fragment maintained its interaction with Glu396 (hydrogen bonding distance of 2.8 Å) and Trp399 (π–π stacking distance of pyridine to indole 3.3 Å with an angle of 94°; Figure 3 B and Supporting Information Figure S2). Comparing the lysine of **12** with that of **11** and TPX2, there is closer alignment with TPX2, as the lysine amine makes hydrogen bonds to the carbonyls of Val321 and Asn361 and the hydroxy group of Thr328 rather than the carbonyl of Gly323. In addition, the interactions with the peptide backbone made by the side chains of Asn361 and Trp357 are the same as observed for the peptide. However the phenylalanine of **12** is poorly defined and appears to be in a hydrophobic pocket defined by Lys353 and Glu354 as opposed to forming π–cation stacking interactions with Arg315 as for the aromatic residue of TPX2. These differences in the alignment of the peptide backbone of **12** compared with the TPX2 represent a potential explanation for its relatively modest affinity (Figure 3 B).

### Removal of the phenyl ring of the biaryl scaffold

**Figure 4:**
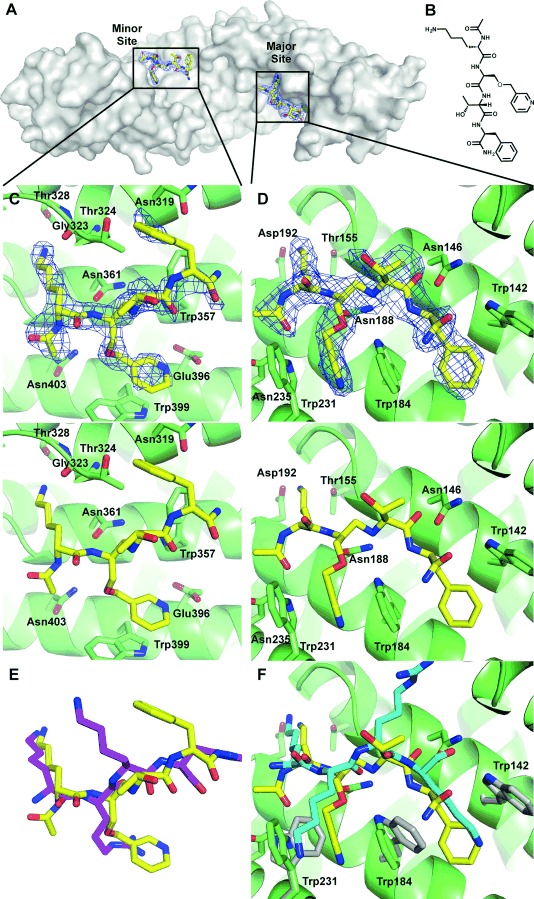
Compound 13 bound to importin-α minor and major sites. A) Compound 13 (yellow) with the 2*F*_o_−*F*_c_ density shown contoured around the ligand at 1.0 *σ* (blue mesh) bound to both the minor and major sites of importin-α (grey surface). B) Structure of compound 13. C), D) Compound 13 (yellow) with the 2*F*_o_−*F*_c_ density bound to the importin-α minor (C) and major (D) sites (top); ligand bound in minor (C) and major (D) sites without density (bottom). E) Compound 13 structure in minor site overlaid with the key TPX2 residues (magenta) from the TPX2–importin-α crystal structure 3KND.[14] F) Compound 13 structure overlaid with the key SV40 residues (cyan) from the SV40–importin-α structure, 1EJL.[30] Grey residues show the positions of the tryptophans in the SV40 structure.

To explore the effect of changing the biaryl fragment scaffold, an unnatural amino acid incorporating a pyridine was synthesised and coupled to give the peptidic KXTF **13**. Direct ITC of **13** gave a *K*_d_ value of 3.9 mm, analogous to **12**. The 2.1 Å resolution structure of **13** bound to importin-α revealed a much better structural overlay of the peptide backbone with that of TPX2 than **12**, with the exception of the phenylalanine that remains poorly defined. As with previous compounds, the pyridine maintains the interactions with Glu396 (hydrogen bond 3.0 Å) and Trp399 (π–π stacking distance of pyridine to indole 3.2 Å with an angle of 89°), even without the biaryl scaffold (Figure 4 C).

Strikingly, for the first time in this study, analysis of the major site showed clear density for another molecule of **13** binding (Figure 4 A). The interaction at the major site involved inducing a 61° rotation of Trp184 and a 137° rotation of Trp231, forming a cryptic pocket with a depth of ∼2.7 Å (Figure 4 D). The pyridine of **13** fills this new cryptic pocket forming face-to-face π–π stacking interactions with Trp184 and Trp231, at distances of 3.3 and 3.4 Å, respectively (Figure 4 D). The rotation of Trp184 also opens a hydrophobic ‘shelf’ on which the phenylalanine of **13** is positioned to form edge-to-face interactions with Trp184 (3.6 Å) and Trp142 (3.7 Å). This result was highly surprising, as no aromatic or hydrophobic containing peptides have been found to bind the major site without long chains of basic flanking residues either side of the central tetrapeptide, with classical major site cargoes having NLSs typified by the SV40 sequence (Figure 4 F).[21] In addition, this suggested that the biaryl scaffold was a source of selectivity for compounds **1**, **11**, and **12**, as its rigidity presumably prevents the pyridine from orienting to open the cryptic pocket as well as introducing another unfavourable aromatic into a polar region of the major site that cannot be masked by other aromatic residues of the protein.

### Linker SAR increases potency and structures identify a new pocket in the minor site

**Figure 5:**
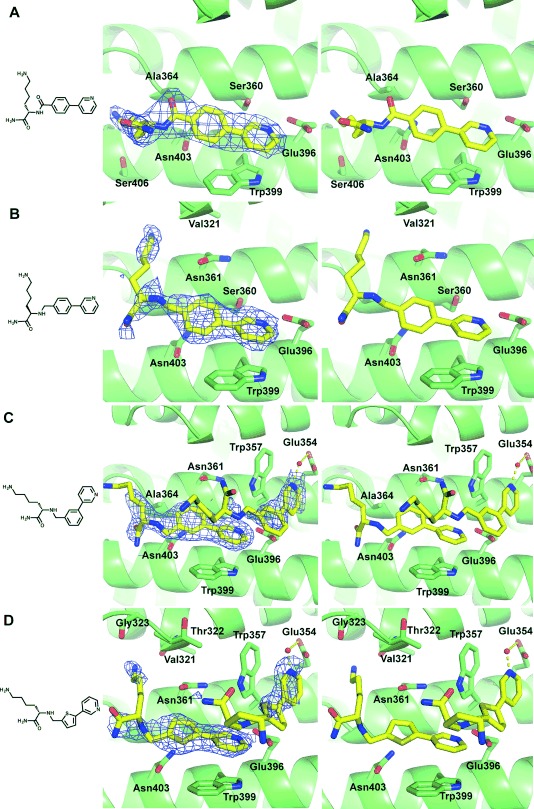
Crystal structures of compounds A) 14, B) 15, C) 16, and D) 17 binding to the importin-α minor site. Left: compound structure; middle: compound (yellow) bound to the minor site of importin-α (green ribbon and sticks) with the 2*F*_o_−*F*_c_ density for each compound contoured around the ligand at 1.0 *σ* (blue mesh); right: compound bound to the minor site without density.

Having established that the biaryl scaffold appears to be the source of selectivity for the minor site in this series of compounds, the crystal structures and docking results were used to design a variety of compounds to explore the structure–activity relationship (SAR) of various fragment–lysine connectivities. The aim was to minimise the strain around the lysine linkage, allowing the backbone flip and improving potency (Table 2). The amide-linked compound **14** had *K*_d_>10 mm by direct ITC, suggesting that its decreased flexibility was deleterious. Changing the stereoisomer **15** of the lysine gave no change in affinity relative to **11**, whereas moving the lysine from the *para* to the *meta* position of the phenyl ring of the biaryl **16** gave a modest improvement in potency. To explore other vectors for the lysine while maintaining similarity to the phenyl, the second ring was replaced with a thiophene (in **17**) resulting in a further increase in potency and the first sub-millimolar compound. X-ray co-crystal structures were solved for all the compounds **14**–**17** bound to importin-α with resolution in the range of 2.0–2.6 Å showing the resultant changes in lysine position and backbone alignment (Figure 5).

**Table 2:** Structures and *K*_d_ values for compounds 11–17.

Compd	Structure	*K*_d_ [mm][a]
**11**	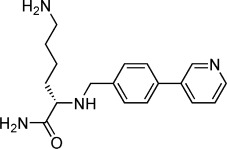	4.0±0.5
**12**	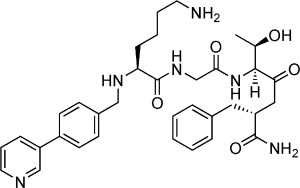	3.3±0.7
**13**	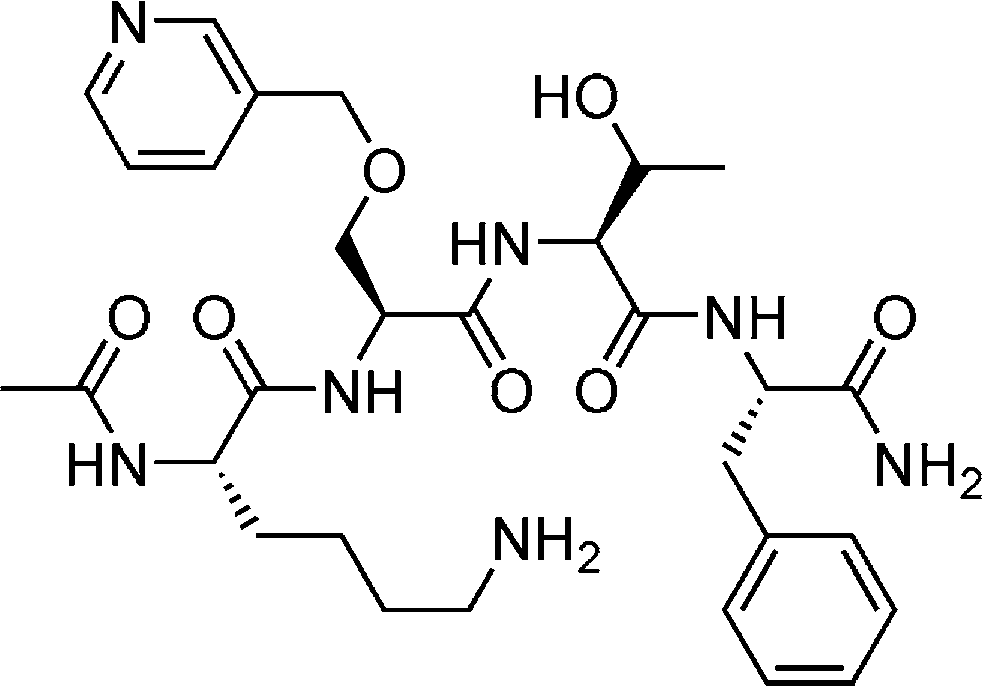	3.9±1.1
**14**	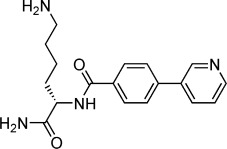	>10.0
**15**	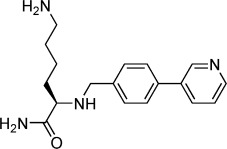	3.8±0.4
**16**	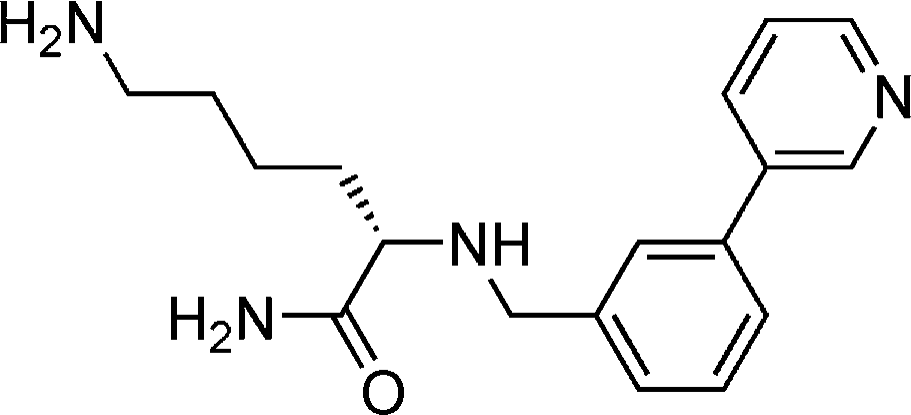	1.3±0.1
**17**	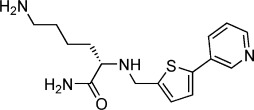	0.9±0.1

[a] Determined by direct ITC at pH 6.0; errors reported are the standard deviations of three replicate runs.

The inclusion of a biaryl scaffold in all cases resulted in selectivity for the minor site, and overlay with the original fragment **1** suggested that these remained ‘anchors’ for the Glu396 interaction despite the changes to the lysine (Supporting Information Figure S2). Both **16** and **17** showed binding in a second pocket of the minor site with an extra compound forming a π–π stacking interaction with Trp357 (3.4 and 4.0 Å, respectively) and a water-mediated hydrogen bond to Glu354 (2.4 Å to water then 2.9 Å to glutamate, and 2.0 Å to water then 2.8 Å to glutamate, respectively), in addition to binding in the Glu396 pocket. In both cases the density for the lysine portion of this second compound was unresolved. For the ligands interacting with Glu396, although the fragment portions of both **16** and **17** overlay well, the lysines behave quite differently. While the lysine amine of **17** forms hydrogen bonds with the carbonyls of Val321 (2.9 Å) and Gly323 (3.2 Å) in the lysine pocket, that of **16** does not reach into the lysine pocket, but instead reorients toward the solvent due to the interaction of the amide with the side chain of Asn403 via a water-mediated hydrogen bond (2.4 Å then 3.5 Å). Such changes perhaps explain the small differences in potency observed between these compounds. Despite the changes in interaction, the increases in affinity remain modest and suggest that the lysine may not make as great a contribution to affinity as was indicated from previous studies with peptide ligands.[13,21]

Finally, the two compounds of **16** and **17** seen binding in close proximity in the importin-α minor site in their co-crystal structures could permit novel fragment linking or growing strategies, between the two biaryl binding sites, moving away from the more flexible lysine and potentially increasing affinity for the minor site.

## Conclusions

PPIs are potential targets in a wide range of diseases, but targeting interactions that are based on unstructured peptides remains a significant challenge, in addition to the problems associated with achieving sufficient specificity. The potential anticancer target interaction between TPX2 and importin-α provides a platform for a pilot study for the use of fragment-based ligand discovery against such targets. A ligand-observed NMR screen of a thermal-shift-enriched fragment library identified several importin-α binding fragments that appeared to bind in the minor site. Structural studies showed that, despite its low affinity, fragment **1** bound specifically in the minor site of importin-α at the key residue Glu396. Information from this structure, as well as docking studies, facilitated the synthesis of a range compounds to probe the contributions made by different groups to affinity for the minor site binding pocket. It was shown that the biaryl scaffold of the fragment is key to the specificity of the compounds for the minor site over the major site, as well as showing that the lysine pocket of the minor site may not make as large a contribution to binding affinity as was thought from previous peptide-based studies. These findings are important considerations for the further development of probes and inhibitors of the TPX2–importin-α site.

These results provide a proof of concept for the use of a fragment-screening cascade in targeting specific sites on multiple site proteins, even when they are based on the binding of unstructured peptides. We have shown fragments to be useful probes in identifying the key binding interactions of open PPI sites as well as achieving and explaining specificity for one site over another. Furthermore, the structures showed that the fragment portion of all the compounds maintained the key interaction with Glu396 despite changes in the lysine or larger peptide positioning, highlighting the effectiveness of fragments as anchors in key PPI hot spots.

## Experimental Section

**Fragment library design**: The fragment library consisted of 1248 commercial fragments based around a core of compounds from Maybridge, supplemented by a selection of compounds chosen to maximise chemical functionality from commercial suppliers Acros Organics, Apollo Scientific, and Sigma–Aldrich. Fragments were stored as 100 mm solutions in [D_6_]DMSO 96-well stock plates.

**Protein expression and purification**: The protein construct used in this study is a truncated form of *Mus musculus* importin-α1 (KPNA2, Accession code: NM_010655) covering residues 70–529, lacking the N-terminal IBB domain: Δ*IBB*-importin-α.[14,22] The protein has >97 % sequence similarity with the *Homo sapiens* variant (Supporting Information Figure S4). The protein was expressed from an in-house modified pET-30a plasmid (EMD Bioscience) with a TEV cleavable His_6_/S-tag that was cleaved during purification; full details are given in the Supporting Information.

**Thermal shift fragment library enrichment**: The changes in the thermal shift of importin-α caused by fragments were assessed using either a Roche LightCycler with opaque PCR plates and clear plastic sealing film (Roche) or on a Bio-Rad Thermocycler with clear PCR plates and flat cap strips (Bio-Rad). Sypro Orange (5000× solution, Invitrogen) was diluted 1:2000 in standard buffer (50 mm Tris⋅HCl pH 8.0, 200 mm NaCl, 1 mm DTT) before addition of importin-α to a concentration of 2 μm and filling assay wells to a volume of 100 μL. The standard Tris buffer was selected from a buffer screen, as it showed the greatest thermal stabilisation of importin-α. Fragments were screened at a final concentration of 5 mm with 5 % DMSO. The fluorescence readout was monitored from 35–60 °C (LightCycler) or 25–60 °C (Thermocycler), both at 0.01 °C s^−1^. All samples were measured in duplicate. Fragments with a negative thermal shift greater than twice the negative standard deviation of each plate’s negative controls wells (5 % DMSO) were removed from the enriched library.

**Ligand-observed NMR screening**: The three ligand-observed techniques—STD, CPMG, and WaterLOGSY—were performed on either a Bruker Avance 500 MHz with TCI cryoprobe or on a Bruker Avance 700 MHz spectrometer with a TXI cryoprobe. All samples were made to a total volume of 200 μL and added to 3 mm capillary tubes (Bruker) before inserting into standard or thick-walled NMR tubes (Bruker). All samples were made to volume in standard protein buffer with 10 % D_2_O and 20 μm TSP. Screens were conducted with fragments at 2 mm from [D_6_]DMSO stock plates, giving a final DMSO concentration of 2 %. Binding was determined on observation of a change in fragment signals in the presence of 20 μm importin-α, relative to a control sample containing only buffer and no protein. If found to bind importin-α, a displacer sample was made by addition of 40 μm TPX2 (IIKPFNLSKGKKRTFDEAAS) peptide (Designer Bioscience) direct to the protein sample and displacement noted if the fragment signals showed a return to those seen in the control sample.

**Isothermal titration calorimetry**: All experiments were performed on a Microcal ITC200 instrument (GE Healthcare) at 25 °C. All ITCs were performed at pH 6.0 using ITC buffer (100 mm citrate pH 6.0, 200 mm NaCl, 10 % DMSO). Fragment ITCs were performed using the competitive ITC technique following the procedure and formula described by Zhang et al.[20] (Supporting Information). Briefly, the titration of 200 μm 20-amino acid TPX2 to 25 μm importin-α 10 % DMSO at pH 6.0 (average *K*_d_=1.0 μm) was compared directly with the same titration in the presence of the fragment at 5 or 10 mm. Direct ITCs were performed for elaborated compounds at pH 6.0 in ITC buffer titrating 10 mm compound into 25 μm protein. Compounds that showed double binding in crystal structures (**13**, **16**, and **17**) had their ITCs fitted using a one-site binding model, as this gave the best curve fitting, suggesting the second binder remains relatively weak. Experimental parameters are described fully in the Supporting Information.

**Crystallisation and crystallographic methods**: Crystals of unliganded Δ*IBB*-importin-α were grown using hanging-drop vapour diffusion in 24-well Linbro plates (Hampton Research) with 4 μL drops of 100 μm Δ*IBB*-importin-α1 at 1:1 protein/precipitant volume ratio (optimal precipitant conditions: 0.1 m citrate pH 6.0, 1.05–1.20 m ammonium sulfate, 20–40 mm DTT) and streak-seeding from an Δ*IBB*-importin-α seed crystal plate 18 h after sealing the wells. Crystals reached their maximum size after 1–2 weeks, when they were soaked in solutions of fragment (100 mm or saturating concentration) in crystallisation buffer supplemented with 10 % DMSO for between 30 min and 5 h. Crystals were then briefly exposed to crystallisation buffer (with half fragment concentration and an additional 10 % glycerol) as a cryoprotectant and flash cooled at 100 K in liquid nitrogen. X-ray data were collected at the Diamond Light Source or European Synchrotron Radiation Facility. All datasets were processed and reduced using *XDS*[23] and *AIMLESS*.[24] The unit cells of these crystals were sufficiently close to that of murine Δ*IBB*-importin-α1 in the *Xenopus*–mouse TPX2–importin-α structure;[14] PDB ID 3KND) to enable rigid body refinement, after which coordinates were refined using iterative cycles of *PHENIX*,[25] *CCP4*,[26] and *COOT*.[27] Electron density maps were then inspected for difference density corresponding to the soaked ligands, which were then built into the model manually using *COOT*, with restraints generated in *PHENIX*. The resulting models were further refined with *CCP4* or *PHENIX*, as well as adding waters and ions from the crystallisation conditions. The stereochemistry of the structure was finally assessed using MolProbity.[28] Molecular graphics were generated using the PyMOL Molecular Graphics System, version 1.6.0.0, Schrödinger LLC. Data collection and refinement statistics are shown in Table S3 of the Supporting Information. Attempts at co-crystallisation are described in the Supporting Information.

**Compound docking**: All compounds were docked into an unliganded structure of mouse Δ*IBB*-importin-α1. The protein was prepared in Discovery Studio Visualizer (Release 3.5, Accelrys Software Inc., San Diego, CA, USA) by removal of crystallographic waters and addition of hydrogens before selecting the coordinates of the key minor site residue Glu396. Compounds to be docked were loaded as .mol files into Discovery Studio Visualizer where hydrogens and relevant charges were added before the structures were minimised to relax bond lengths and fix bond angles. Ligands were docked into the protein in a radius of 13 Å around Glu396 using GOLD 5.1[29] using default slow docking settings with 25 runs for each ligand. Figures of the ten lowest-energy docked positions of each were exported and subjected to visual analysis.

**Synthetic chemistry**: General directions for the synthesis of the compounds described as well as their spectroscopic analyses are supplied in the Supporting Information. The purity of all compounds tested for binding to importin-α was assessed by LCMS or HPLC and is >95 % unless otherwise stated.

**Accession numbers**: The coordinates and structure factors for the mouse–mouse TPX2–importin-α structure used to inform compound synthesis, as well as importin-α compound structures **1**, **11**, **12**, **13**, **14**, **15**, **16**, and **17** have been deposited in the RCSB Protein Data Bank (http://www.pdb.org) under the PDB ID codes: 4U54, 4U5L, 4U5N, 4U5S, 4U58, 4U5O, 4U5U, and 4U5V respectively.
