# Supplementary material for: Selective Targeting of the TPX2 Site of Importin-α Using Fragment-Based Ligand Design
Source: ChemMedChem. 2015 Apr 20;10(7):1232–9. doi: 10.1002/cmdc.201500014 (PMC4515083; doi:10.1002/cmdc.201500014)
Supplement: Supplementary file 1 [file cmdc0010-1232-sd1.pdf]

CHEMMEDCHEM
Chemistry & Drug Discovery

Supporting Information

Selective Targeting of the TPX2 Site of Importin-α Using Fragment-Based Ligand Design

Rhian S. Holvey,[a] Eugene Valkov,[b] David Neal,[c] Murray Stewart,[b] and Chris Abell*[a]

cmdc_201500014_sm_miscellaneous_information.pdf

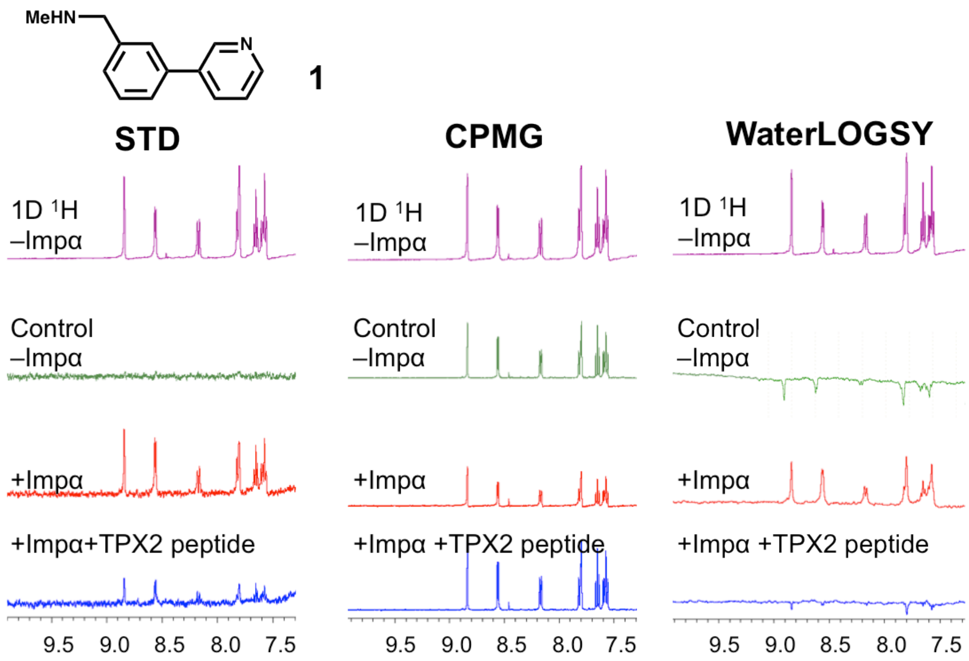

Figure S1. Ligand-observed NMR techniques STD, CPMG and WaterLOGSY illustrating the binding of fragment 1 to importin-α. Top row of each NMR 1D spectrum of Fragment 1; Control -Impα: 2 mM fragment in the absence of the protein; +Impα: 2 mM fragment in the presence of 20 μM protein, changes in signal indicating binding; +Impα +TPX2 peptide: displacement study by addition of 40 μM of the TPX2 peptide suggested binding was to the minor site.

Table S1. Structures of 46 fragments that bind importin-α and are displaced in at least one of STD, WaterLOGSY and CPMG.

No.	Structure	No.	Structure	No.	Structure
1	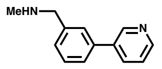	-	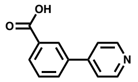	-	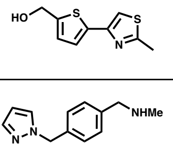
2	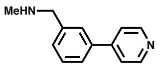	-	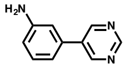	-	
3	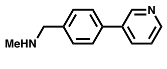	-	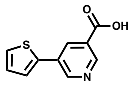	-	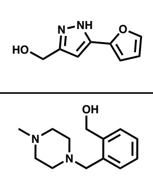
4	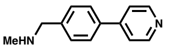	-	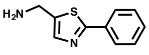	-	
5	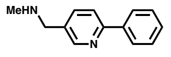	-	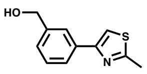	-	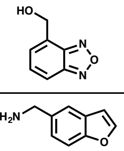
6	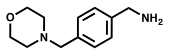	-	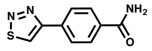	-	
7	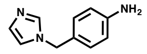	-	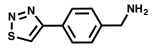	-	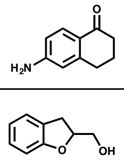
8	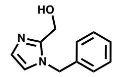	-	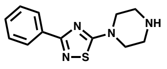	-	
9	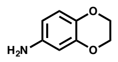	-	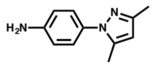	-	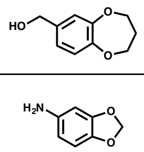
10	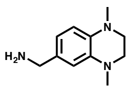	-	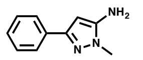	-	
-	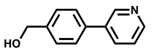	-	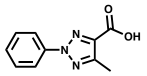	-	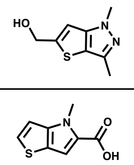
-	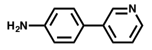	-	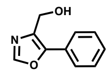	-	
-	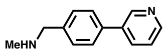	-	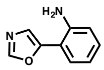	-	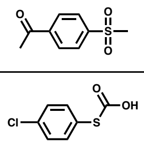
-	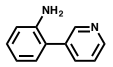	-	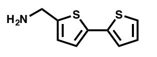	-	
-	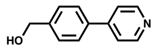	-	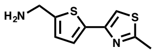	-	

Table S2. Structures and competitive ITC K_d values at pH 8.0 of fragments 1 – 10 that were prioritised for crystallography using their competitive K_d values at pH 6.0 (see Table 1).

Comp.	Structure	K_d pH 8.0 /mM	Comp.	Structure	K_d pH 8.0 /mM
1	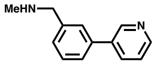	>15	-	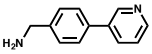	>15
2	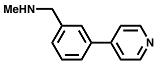	No competition	-	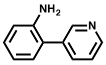	No competition
3	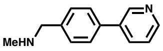	>15	-	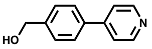	>15
4	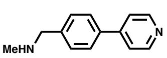	No competition	-	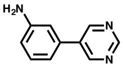	No competition
5	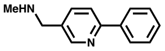	>15	-	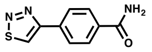	No competition
6	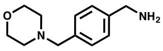	10.0 ± 1.0	-	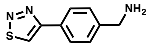	>15
7	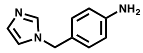	>15	-	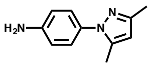	>15
8	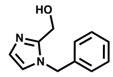	>15	-	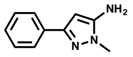	>15
9	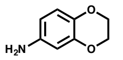	>15	-	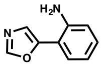	>15
10	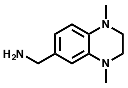	3.2 ± 0.8	-	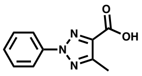	>15
-	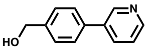	>15	-	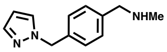	>15
-	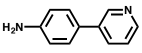	>15	-	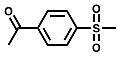	>15

Error reported is compound error from the standard deviations of two replicate control experiments and two replicate competitive fragment experiments.

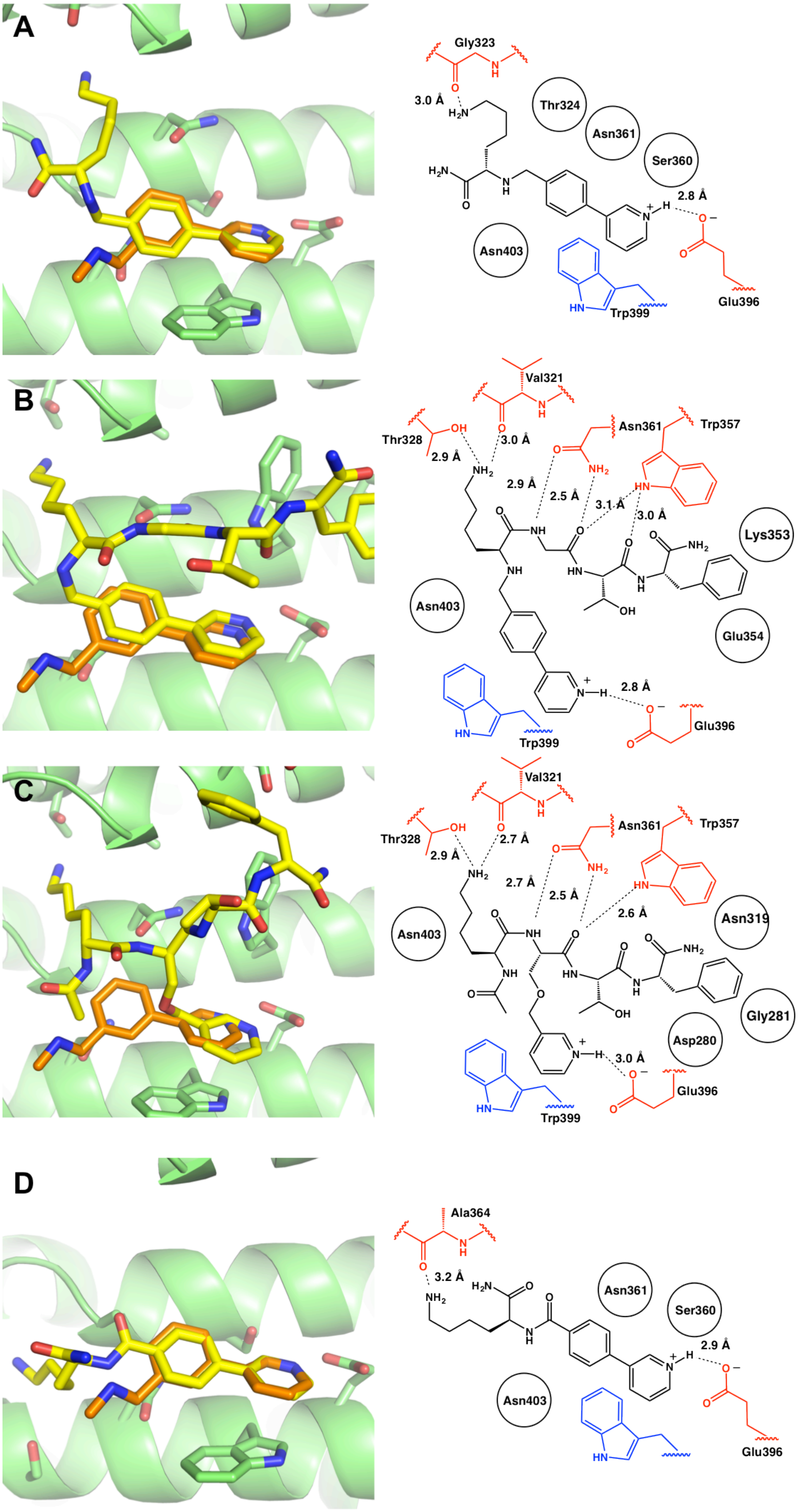
A
Gly323
3.0 Å
Thr324
Asn361
Ser360
2.8 Å
Asn403
Trp399
Glu396
B
Val321
Thr328
Asn361
Trp357
2.9 Å
3.0 Å
2.9 Å
2.5 Å
3.1 Å
3.0 Å
Lys353
Asn403
Glu354
2.8 Å
Trp399
Glu396
C
Val321
Thr328
Asn361
Trp357
2.9 Å
2.7 Å
2.7 Å
2.5 Å
2.6 Å
Asn403
Asn319
Gly281
Asp280
3.0 Å
Trp399
Glu396
D
Ala364
3.2 Å
Asn361
Ser360
2.9 Å
Asn403
Glu396

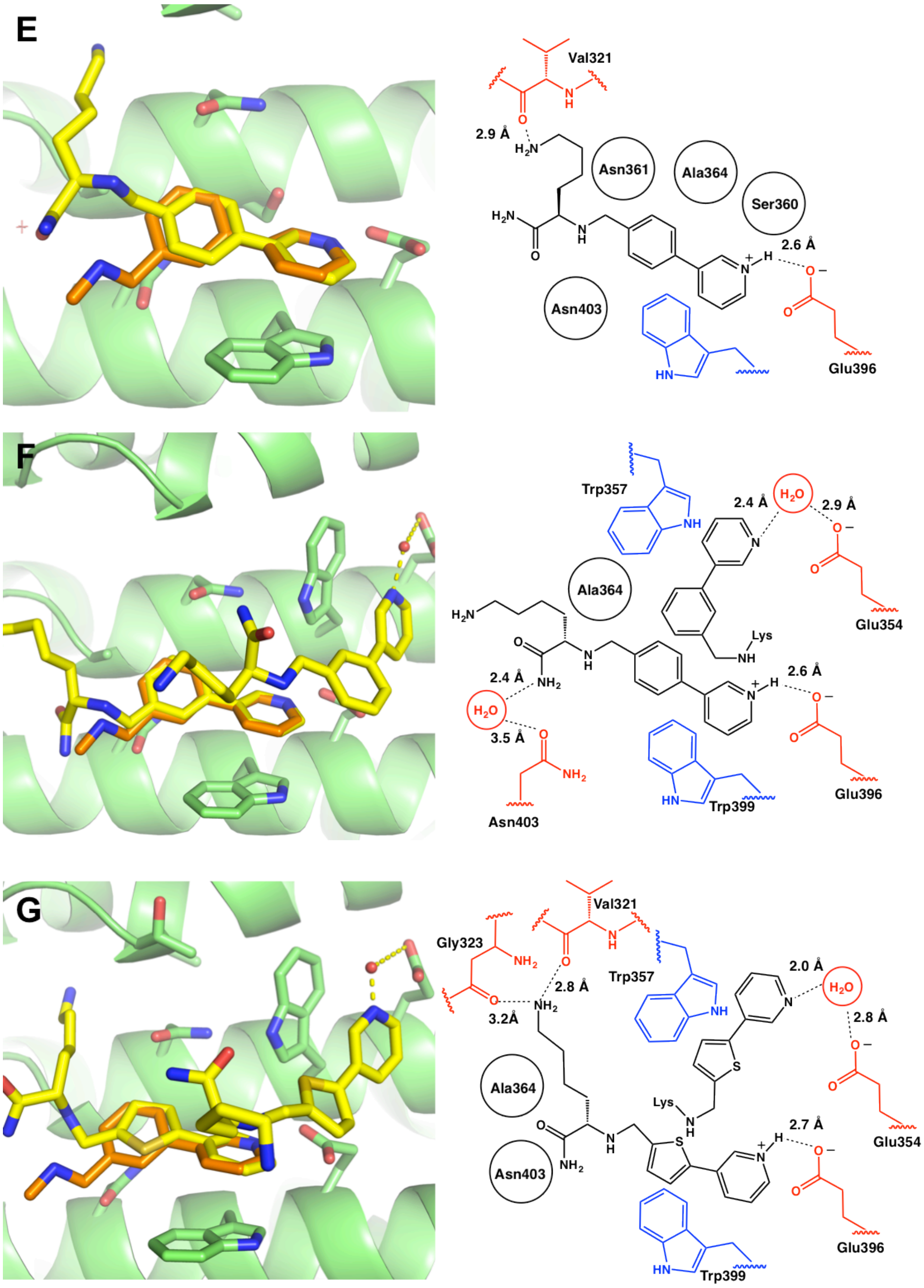

Figure S2. Crystal structures of compounds 11 – 17 compared with fragment 1 and schematic of their key minor site interactions. Overlay of compound (yellow) with fragment **1** (orange) in the minor site of importin-α (green cartoon and sticks) (left); schematic illustrations showing the key interactions with the minor site ligands; red residues are making hydrogen-bonds, or salt-bridge contacts, blue residues are involved in π-π stacking interactions and residues denoted by black circles form non-polar interactions with the ligand (right). (A) Compound **11**, (B) Compound **12**, (C) Compound **13**, (D) Compound **14**, (E) Compound **15**, (F) Compound **16**, (G) Compound **17**.

Table S3. Compound-bound importin-α crystallographic statistics.

**Data Collection Statistics**	**1**	**11**	**12**	**13**	**14**	**15**	**16**	**17**
Wavelength /Å	0.9686	0.9192	0.9763	0.9200	0.9200	0.9763	0.9763	0.9763
Space group	$P2_12_12_1$	$P2_12_12_1$	$P2_12_12_1$	$P2_12_12_1$	$P2_12_12_1$	$P2_12_12_1$	$P2_12_12_1$	$P2_12_12_1$
Unit cell parameters:								
a, b, c, /Å	78.4, 91.3, 98.8	78.9, 90.6, 100.4	78.5, 91.0, 98.9	79.6, 91.3, 102.2	79.1, 90.6, 101.3	78.9, 90.8, 100.5	78.6, 90.4, 100.0	79.0, 90.8, 99.7
α, β, γ /°	90.0, 90.0, 90.0	90.0, 90.0, 90.0	90.0, 90.0, 90.0	90.0, 90.0, 90.0	90.0, 90.0, 90.0	90.0, 90.0, 90.0	90.0, 90.0, 90.0	90.0, 90.0, 90.0
Resolution /Å	20.0 – 2.41 (2.50 – 2.41)*	19.8 – 2.53 (2.64 – 2.53)*	67.0 – 2.3 (2.39 – 2.31)*	20.0 – 2.12 (2.18 – 2.12)*	62.3 – 2.56 (2.63 – 2.56)*	67.4 – 2.0 (2.07 – 2.00)*	67.1 – 1.96 (2.03 – 1.96)*	67.1 – 1.95 (2.02 – 1.95)*
Unique reflections	27,498	24,615	31,713	42,601	23,427	47,192	51,660	49,375
Total observations	119,421	256,218	247,122	363,041	122,842	220,763	250,382	226,173
$\langle I / \sigma(I) \rangle$	11.0 (2.3)	7.9 (2.1)	13.4 (1.8)	17.7 (3.5)	13.8 (2.4)	10.2 (1.8)	14.8 (1.6)	13.6 (1.2)
R_{merge}	0.10 (0.75)	0.13 (0.83)	0.15 (1.49)	0.09 (0.67)	0.12 (0.95)	0.10 (1.08)	0.07 (1.21)	0.07 (1.47)
Completeness /%	98.4 (99.4)	99.7 (99.9)	99.8 (99.8)	99.2 (99.7)	97.5 (99.8)	99.0 (99.9)	99.6 (99.9)	96.8 (89.5)
Multiplicity	4.3	10.4	7.8	8.5	5.2	4.5	4.8	4.3
Wilson B-factor	31.1	50.1	46.9	24.1	29.4	50.3	46.7	48.3
**Refinement statistics**								
No. non-H atoms								
Protein	3,240	3,261	3,254	3,254	3,220	3,254	3,254	3,254
Ligand	15	23	45	88	24	23	46	44
R.M.S. deviations								
Bond lengths /Å	0.010	0.009	0.009	0.009	0.009	0.009	0.008	0.010
Bond angles /°	1.203	1.217	1.254	1.281	1.242	1.238	1.267	1.657
Average B-factor /$Å^2$	40.2	34.1	50.8	39.4	44.1	38.3	42.7	53.4
Ramachandran favoured/ outliers /%	98.1/0.0	96.3/0.7	97.1/0.2	99.3/0.0	98.1/0.0	98.4/0.0	97.9/0.5	97.9/0.2
All-atom clashscore	5.3	5.9	3.3	3.4	6.1	6.4	5.9	8.6
Random reflections	1,384	1,256	1,597	2,125	1,198	2,355	2,634	2,483
R_{work}/R_{free} /%	18.6/22.7	18.2/22.2	18.7/22.1	19.8/23.1	21.4/26.4	21.7/24.7	19.5/21.9	22.7/25.8
MolProbity score	1.28	1.56	1.27	1.12	1.38	1.33	1.36	1.49
Coordinate error	0.30	0.26	0.27	0.21	0.32	0.26	0.22	0.26
PDB accession code	4U54	4U5L	4U5N	4U5S	4U58	4U5O	4U5U	4U5V

All data was collected at 100 K. *Values in parentheses are for highest-resolution shell.

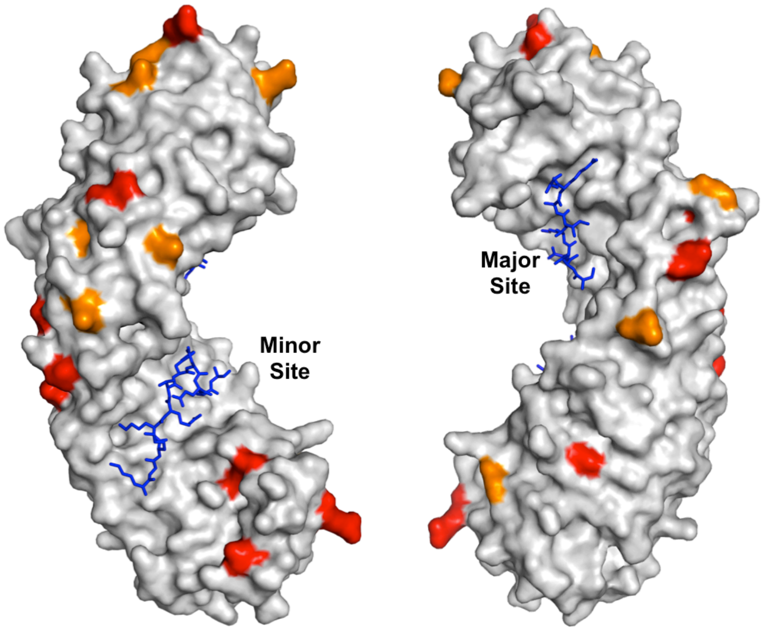

Figure S3. Comparison of human and mouse KPNA2 shown on mouse TPX2$^{(305-370)}$ – importin-α crystal structure. Grey residues are identical, orange are conserved and red are changed. Blue sticks are TPX2, from the TPX2 – importin-α structure 3KND.[1]

Supplemental Experimental Procedures

Supplemental protein expression and purification

Plasmids were transformed into *E. coli* BL21 (DE3) CodonPlus RIL competent cells[2] by heat shock at 42 °C, 1 min, 0 °C, 5 min and then incubation in 1 mL 2xTY media at 37 °C, 30 min. These were then plated on TYE (Kanamycin 25 µg/mL) agar plates and grown overnight at 37 °C. Single colonies were then grown in starter cultures of 2× 50 mL LB media (Kanamycin 25 µg/mL) on a shaker at 37 °C overnight. From these 20x 1 L 2xTY expression media (Kanamycin 25 µg/mL) were inoculated at 1:100 volume dilution and grown at 37 °C to an OD_{600} of 0.6 – 0.8. The temperature was lowered to 20 °C and overnight expression was induced with IPTG (1 mM final concentration). Cells were harvested by centrifugation (5,000 rpm, 15 min, 4 °C) and pellets were either frozen at -20 °C for future purification or were resuspended in lysis buffer (20 mM Tris.HCl pH 8.0, 200 mM NaCl, 2 mM β-mercaptoethanol).

To resuspended cell pellets were added PMSF (1 mM) and 2 anti-protease tablets (Roche) before lysis by sonication while keeping samples on ice. 0.04% Bovine DNase I (Sigma Aldrich), 0.1% Mn^{2+} and 1% Mg^{2+} were then added to the lysate on ice, before separation of the cell debris by centrifugation (20,000 rpm, 30 min, 4 °C). Further clarification was performed through 0.22 µm syringe filters (Sartorius) and these clarified lysates were added to a glass column containing Nickel NTA agarose resin (Qiagen, 2.5 mL of 50% slurry per litre of culture) pre-equilibrated with buffer (20 mM Tris.HCl pH 8.0, 200 mM NaCl, 2 mM β-mercaptoethanol, 15 mM imidazole) and incubated under gentle agitation at room temperature for 1 hr. The affinity resin was washed extensively with lysis buffer (4× 10 fold excess of resin) before eluting with 2× 10-fold excess of resin of 20 mM Tris.HCl pH 8.0, 200 mM NaCl, 2 mM β-mercaptoethanol, 300 mM imidazole. The eluent was then added to a dialysis tube with 1:40 molar ratio tobacco etch virus (TEV) protease and 0.5 mM DTT and placed

in a beaker of elution buffer (50 mM Tris.HCl pH 8.0, 200 mM NaCl, 1 mM DTT) overnight at 4 °C.

Further purification of concentrated protein was conducted by size exclusion chromatography on a HiLoad 26/60 Superdex-200 prep grade column (GE Healthcare) connected to an ÄKTAexplorer (GE Healthcare) and eluted with standard protein buffer: 50 mM Tris.HCl pH 8.0, 200 mM NaCl, 1 mM DTT. The buffer flow rate was set at 2 mL/min, fraction sizes at 5 mL and all size exclusion chromatography work was performed at 4 °C.

The purified protein fractions were collected and analysed for purity by SDS-PAGE (using standard PAGE protocols) and those deemed to be >95% pure were pooled and concentrated to 200 µM (as assessed by Nanodrop) using 30 kDa cut-off Amicon Ultra concentrator tubes (Millipore). Average protein yield was ~10 mg/L and mass spectrometry showed a molecular weight of 50435 ± 90 Da consistent with untagged ΔIBB-importin-α1. The protein was flash-frozen in liquid nitrogen in 100 or 200 µL aliquots in thin-wall PCR tubes before storing at -80 °C. Each aliquot was defrosted fully prior to use.

Isothermal titration calorimetry competitive ITC formula and run parameters

Competitive ITC K_as were calculated using Formula S1 described by Zhang *et al.*:

$$\text{Formula S1:}\quad K_{a2}=\left(\frac{K_{a1}}{K_{aApp}}-1\right)\frac{1}{L_{2Tot}}$$

Where K_{a2} is the desired K_a ($K_a = 1/K_d$), K_{a1} the K_a of the peptide, K_{aApp} the apparent K_a of the peptide in the competition experiment and L_{2Tot} the total concentration of the competitor or fragment.

All experiments, competitive and direct, were run using the optimised experiment conditions: 20 injections (first volume 1 µL subsequent 2 µL with default durations), reference power 5 – 7 µCal/sec, initial delay 60 sec, stirring speed 1000 rpm, spacing 90 sec and filter period of 2 sec. Experiment injection of 39 µL titrant into 250 µL of protein solution over 40 min.

Co-crystallisation attempts

A screen for co-crystallisation conditions of unliganded mouse ΔIBB-importin-α1 with fragments **1** – **10** was set up in the MRC-LMB using in-house robotic nanolitre crystallisation methods on 96-well plates as described by Stock *et al.* [3]. The protein was used in the purification buffer at 200 µM supplemented with 10% DMSO and 20 – 50 µM of the desired fragment (as determined by solubility). 200 nL drops were set up as a 1:1 protein to precipitant volume ratio on in-house 96 well plates with a range of crystallisation conditions. No crystal hits were seen in any case even after three months at 18 °C.

Synthetic Chemistry

Standard Procedure A) Solid phase peptide synthesis

Solid phase peptide synthesis was performed in batches on NovaPEG Rink amide resin (Novabiochem, Merck Chemicals Ltd.) that was swelled with DCM and then DMF before deprotection and coupling steps. The following commercial protected amino acids were used: Fmoc-L-Arg(Pbf)-OH, Fmoc-L-Gly-OH, Fmoc-L-Lys(Boc)-OH, Fmoc-L-Thr(^{t}Bu)-OH, and Fmoc–L-Phe-OH (AGTC Bioproducts or Merck Novabiochem). Removal of the Fmoc protecting group at the start of each coupling was performed with 3× 20% piperidine in DMF (v/v) for 5 min each. The resin was then washed with 5× DMF before addition of 5 eq. of Fmoc-protected amino acid, 5

eq. PyBOP and 10 eq. DIPEA in DMF. The vessel was then sealed and left on a shaker at room temperature for the time described. Couplings were repeated until a Kaiser Test on a small portion of the resin was negative. Further deprotections and couplings were repeated as above.

On coupling and deprotection of the final amino acid, peptides were either capped by *N*-terminal acetylation or coupled to a fragment containing monomer. *N*-terminal acetylation was affected by 1 eq. of acetic anhydride in DMF for 20 min while fragment coupling was performed using 5 eq. of the fragment containing monomer and either 5 eq. PyCLOCK or PyBOP and 10 eq. DIPEA in DMF or NMP under the conditions and times described.

Once capped, the finished peptide was washed with 5× DMF, 5× DCM and dried before cleaving with concomitant global deprotection with 4 mL 95% TFA in water (v/v) for 3 hr at room temperature. Resin was washed with a further 2 mL 95% TFA in water before the majority of TFA was evaporated under a nitrogen stream. The peptide was precipitated with ice-cold diethyl ether overnight and collected by centrifugation before washing with 3× cold diethyl ether to give the crude peptide which was then lyophilised overnight before purification by HPLC. Product peaks were isolated and lyophilised overnight giving the product.

Standard Procedure B) Suzuki couplings

1 eq. of each boronic acid and bromide starting material were combined in a microwave vial (Biotage) and dissolved in DME before the addition of potassium carbonate (2 eq.) dissolved in water (3:1 v/v DME/water). Nitrogen was bubbled through the stirring solution before the addition of Pd(dppf)Cl_2 (0.04 eq.) and sealing the vial. The reaction was heated in a Biotage Initiator Microwave Synthesizer at 120 ^{o}C for 2 hr unless otherwise stated. The majority of DME was removed *in vacuo*

before addition of sat. sodium bicarbonate solution and extraction with 3× EtOAc. The combined organics were then dried with anhydrous magnesium sulphate, filtered and the solvent was removed *in vacuo* to give the crude compound, which was then purified as described.

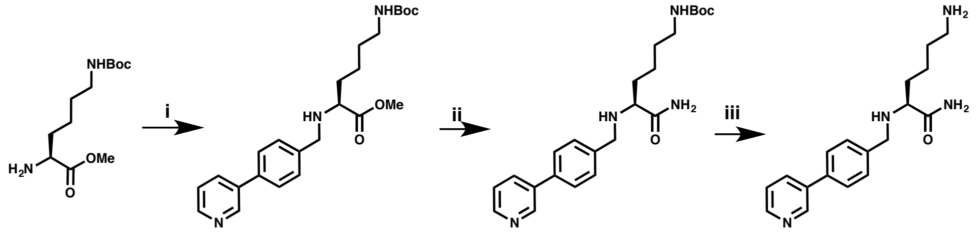

11

40% 55% 49%

i 1.03 eq 4-(pyridin-3-yl)benzaldehyde, 1.1 eq NaBH(OAc)$_3$, THF, 18 hr.
ii 5 eq Mg_3N_2, MeOH, 80 °C, 24 hr.
iii 4 M HCl in dioxane, 3 hr.

Methyl N^6-(tert-butoxycarbonyl)-N^2-(4-(pyridin-3-yl)benzyl)-L-lysinate [4]

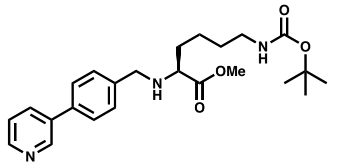

H-Lys(Boc)-OMe.HCl (500 mg, 1.60 mmol) and 4-(pyridine-3-yl)benzaldehyde (302 mg, 1.65 mmol) were combined in THF (12 mL) before addition of sodium triacetoxyborohydride (371 mg, 1.76 mmol) and stirring at room temperature for 18 hr. The reaction was quenched with sat. potassium carbonate (15 mL) for 1 hr before extraction with EtOAc (3× 15 mL). The combined organics were washed with brine (15 mL), dried over anhydrous magnesium sulphate, filtered and reduced *in vacuo* to give the crude product. Further purification affected by column chromatography (Biotage, eluent 0 – 15% MeOH/DCM v/v) to give *pyridine methyl lysinate* as a yellow oil (275 mg, 40% yield).

R_F (10% MeOH in DCM v/v) = 0.4. $\nu_{max.}$ 1745 cm^{-1} (C=O); ^{1}H NMR (500 MHz, $CDCl_3$) δ 8.84 (1H, d, *J* 1.8 Hz), 8.58 (1H, dd, *J* 1.3, 4.7 Hz), 7.87 (1H, ddd, *J* 1.7, 2.3, 7.9 Hz), 7.54 (2H, dt, *J* 1.9, 8.2 Hz), 7.44 (2H, d, *J* 8.4 Hz), 7.36 (1H, ddd, *J* 0.8, 4.8, 7.9 Hz), 4.52 (1H, br. s), 4.37 (1H, s), 3.87 (1H, d, *J* 13.2 Hz), 3.74 (3H, s), 3.67

(1H, d, *J* 13.2 Hz), 3.28 (1H, t, *J* 6.7 Hz), 3.13 – 3.07 (2H, m), 1.74 – 1.57 (2H, m), 1.52 – 1.38 (13H, m); ^{13}C NMR (125 MHz, $CDCl_3$) δ 175.8, 155.9, 148.3, 148.2, 139.9, 136.6, 136.4, 134.3, 129.0, 122.1, 123.6, 79.1, 76.8, 60.6, 51.7, 40.3, 33.1, 29.8, 28.4, 23.0; LCMS $[M+H]^+$ 428.2, R_t = 3.1 min; HRMS calc. for $C_{24}H_{34}N_3O_4$ MH^+: 428.2549. Found MH^+: 428.2578.

Tert-butyl (S)-(6-amino-6-oxo-5-((4-(pyridin-3-yl)benzyl)amino)hexyl)carbamate [5]

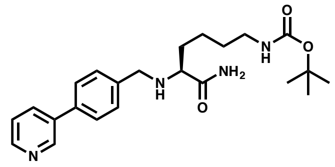

A solution of Methyl N^6-(*tert*-butoxycarbonyl)-N^2-(4-(pyridin-3-yl)benzyl)-L-lysinate (303 mg, 0.71 mmol) was made up in a microwave vial (Biotage) with methanol (4 mL) before cooling to 0 °C whereupon magnesium nitride (356 mg, 3.55 mmol) was added in one portion. The vial was sealed and allowed to warm to room temperature in a water bath for 1 hr. The reaction was then heated to 80 °C for 24 hr, carefully monitoring the state of the cap over the first 3 hr. CAUTION: these reactions must be carried out behind a blast shield. The reaction was cooled to room temperature and vented carefully before diluting with chloroform (25 mL) and water (25 mL). The reaction was neutralised with HCl (3N) before the layers were separated and the aqueous layer further extracted with chloroform (3× 25 mL). The combined organics were dried over anhydrous magnesium sulphate, filtered and reduced *in vacuo*. The crude product was purified by column chromatography (Biotage, eluent 0 – 20% MeOH/DCM v/v) to give the *pryridine lysine amide* as a pale yellow solid (159 mg, 55% yield).

R_F (10% MeOH in DCM v/v) = 0.4. $\nu_{max.}$ 3383, 3346 cm^{-1} (N-H), 1675 cm^{-1} (C=O); ^{1}H NMR (400 MHz, $CDCl_3$) δ 8.80 (1H, s), 8.58 (1H, dd, *J* 1.1, 3.8 Hz), 7.85 (1H, ddd, *J* 1.4, 1.8, 6.3 Hz), 7.56 – 7.52 (2H, m), 7.47 – 7.43 (2H, m), 7.36 (1H, ddd, *J* 0.5, 3.9, 6.3 Hz), 7.20 (1H, s), 5.68 (1H, s), 4.61 (1H, s), 3.90 (1H, d, *J* 10.6 Hz),

3.81 (1H, d, *J* 10.6 Hz), 3.32 – 3.24 (1H, m), 3.15 – 3.04 (2H, m), 1.86 – 1.67 (2H, m), 1.50 – 1.36 (13H, m); ^{13}C NMR (100 MHz, $CDCl_3$) δ 176.2, 156.1, 148.5, 148.2, 138.4, 137.1, 136.1, 134.3, 129.1, 127.3, 123.6, 79.2, 62.0, 53.4, 40.0, 32.7, 30.9, 28.4, 22.7; LCMS $[M+H]^+$ 413.6, R_t = 3.1 min; HRMS calc. for $C_{23}H_{33}N_4O_3$ MH^+: 413.2553. Found MH^+: 413.2578.

(S)-6-Amino-2-((4-(pyridin-3-yl)benzyl)amino)hexanamide ***11***

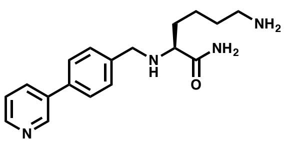

Tert-butyl (*S*)-(6-amino-6-oxo-5-((4-(pyridin-3-yl)benzyl)amino)hexyl)carbamate (170 mg, 0.41 mmol) was dissolved in 4 M HCl in dioxane (15 mL) and stirred for 3 hr at room temperature. The reaction was reduced *in vacuo* to give the crude compound, which was further purified by HPLC as described in Section 5.5.1. Product fractions eluted at 9 min and were lyophilised to afford the TFA salt of the *pyridine lysine amide* ***11*** as an orange solid (63 mg, 49% yield).

HPLC elution at 9 min, purity >95%; $\nu_{max.}$ 3400 cm^{-1} (N-H), 1698 cm^{-1} (C=O); ^{1}H NMR (500 MHz, d_6-DMSO) δ 9.11 (1H, d, *J* 1.6 Hz), 8.75 (1H, d, *J* 4.5 Hz), 8.48 (1H, d, *J* 8.1 Hz), 7.98 (2H, s), 7.91 – 7.87 (2H, m), 7.80 (1H, dd, *J* 5.2, 8.0 Hz), 7.77 – 7.70 (2H, m), 7.30 (1H, s), 6.69 (1H, s), 4.44 – 4.20 (2H, m), 3.78 – 3.72 (1H, m), 2.83 – 2.72 (2H, m), 1.98 – 1.80 (2H, m), 1.66 – 1.52 (2H, m), 1.49 – 1.29 (2H, m); ^{13}C NMR (125 MHz, d_6-DMSO) δ 171.5, 145.5, 144.4, 138.1, 136.3, 132.3, 132.2, 131.2, 127.3, 125.5, 58.8, 48.6, 38.2, 29.0, 26.6, 21.3; ^{19}F (376 Hz, d_6-DMSO) δ -73.9, -74.3; LCMS $[M+H]^+$ 313.1, R_t = 0.8 min; HRMS calc. for $C_{18}H_{25}N_4O$ MH^+: 313.2028. Found MH^+: 313.2032; $[\alpha]_D^{25}$ = +2.7.

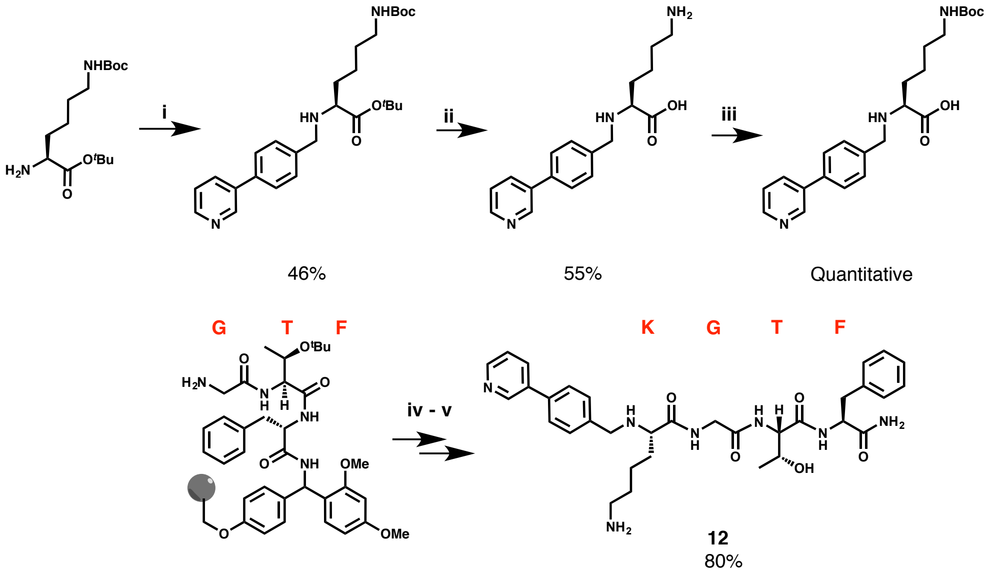

i 1.03 eq 4-(pyridin-3-yl)benzaldehyde, 1.1 eq NaBH(OAc)$_3$, THF, 18 hr.
ii 20% TFA in DCM, 8 hr.
iii $(Boc)_2O$, 10% NEt_3 in MeOH, 30 min, 60 °C.
iv 5 eq cap, 5 eq PyClock, 10 eq DIPEA, DMF, 3.5 hr.
v TFA: H_2O 95: 5, 4 hr.

Tert-butyl N^6-(tert-butoxycarbonyl)-N^2-(4-(pyridin-3-yl)benzyl)-L-lysinate [4]

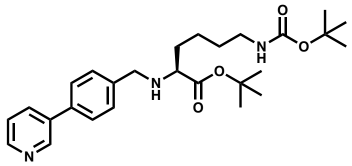

H-Lys(Boc)-O^{t}Bu (186 mg, 0.55 mmol), 4-(pyridine-3-yl)benzaldehyde (100 mg, 0.55 mmol) and acetic acid (40 μL, 1% v/v) were combined in THF (4 mL) before addition of sodium triacetoxyborohydride (129 mg, 0.61 mmol) and stirring at room temperature for 18 hr. The reaction was quenched with sat. potassium carbonate (5 mL) and stirred for 1 hr before extraction with EtOAc (3× 10 mL). The combined organics were washed with sat. sodium bisulphite solution (3× 10 mL), dried over anhydrous magnesium sulphate, filtered and reduced *in vacuo* to give the crude product. Further purification affected by column chromatography (Biotage, eluent 0 – 10% MeOH/DCM v/v). Product fractions reduced *in vacuo* to give the *pyridine lysinate* as a colourless oil (118 mg, 46% yield).

R_F (10% MeOH in DCM v/v) = 0.5; $\nu_{max.}$ 3356 cm^{-1} (N-H secondary), 1708 cm^{-1} (C=O), 1248 cm^{-1} (C-O), 1153 cm^{-1} (C-N); ^{1}H NMR (500 MHz, CD_3OD)

δ 8.79 (1H, dd, *J* 0.6, 2.2 Hz), 8.51 (1H, dd, *J* 1.5, 4.9 Hz), 8.06 (1H, ddd, *J* 1.6, 2.3, 8.0 Hz), 7.64 – 7.61 (2H, m), 7.51 – 7.46 (3H, m), 3.85 (1H, d, *J* 13.0 Hz), 3.71 (1H, d, *J* 13.1 Hz), 3.17 (1H, t, *J* 6.7 Hz), 3.04 (2H, t, *J* 6.7 Hz), 1.72 – 1.60 (2H, m), 1.53 – 1.35 (22H, m); ^{13}C NMR (125 MHz, CD_3OD) δ 175.5, 158.4, 148.7, 148.2, 140.9, 138.2, 137.4, 136.3, 130.5, 128.1, 125.4, 82.4, 79.7, 62.0, 52.3, 41.0, 33.8, 30.7, 28.8, 28.4, 23.9; LCMS $[M+H]^+$ 470.0, R_t = 3.5 min; HRMS calc. for $C_{27}H_{40}N_3O_4$ MH^+: 470.3019. Found MH^+: 470.3018.

(4-(Pyridin-3-yl)benzyl)-L-lysine

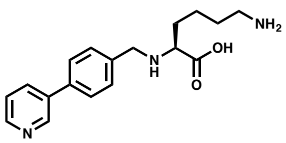

Tert-butyl *N*^{6}-(*tert*-butoxycarbonyl)-*N*^{2}-(4-(pyridin-3-yl)benzyl)-L-lysinate (88 mg, 0.19 mmol) was dissolved in DCM (2.4 mL) before TFA (600 µL, 20% v/v) was added and the reaction stirred at room temperature for 8 hr. The solvents were removed *in vacuo* to give a TFA salt of the *pyridine lysine* as a pale yellow powder (66 mg, quantitative).

^{1}H NMR (500 MHz, CD_3OD) δ 9.04 (1H, d, *J* 2.0 Hz), 8.73 (1H, dd, *J* 1.4, 5.3 Hz), 8.57 (1H, ddd, *J* 1.5, 2.2, 8.2 Hz), 7.92 – 7.86 (3H, m), 7.74 – 7.70 (2H, m), 4.36 (2H, s), 4.02 (1H, dd, *J* 5.0, 7.7 Hz), 2.95 (2H, t, *J* 7.7 Hz), 2.12 – 1.97 (2H, m), 1.80 – 1.68 (2H, m), 1.67 – 1.50 (2H, m); ^{13}C NMR (125 MHz, CD_3OD) δ 171.1, 145.4, 144.7, 141.3, 139.4, 138.1, 133.5, 132.4, 129.1, 127.3, 61.0, 51.1, 40.3, 30.3, 28.1, 23.2; LCMS $[M+H]^+$ 314.0, $[M-H]^-$ 312.1, R_t = 0.6 min; HRMS calc. for $C_{18}H_{24}N_3O_2$ MH^+: 314.1869. Found MH^+: 314.1852.

(S)-6-Amino-N-(2-(((2S,3R)-1-(((S)-1-amino-1-oxo-3-phenylpropan-2-yl)amino)-3-hydroxy-1-oxobutan-2-yl)amino)-2-oxoethyl)-2-((4-(pyridin-3-yl)benzyl)amino) hexanamide ***12***

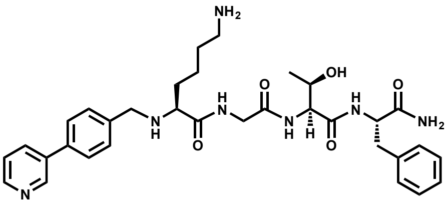

N^6-(*tert*-butoxycarbonyl)-*N*^{2}-(4-(pyridin-3-yl)benzyl)-L-lysine by combining (4-(Pyridin-3-yl)benzyl)-L-lysine (66 mg, 0.19 mmol) with Di-*tert*-butyl dicarbonate (87 μL, 0.38 mmol) in methanol (5 mL) with 10% triethylamine and heating for 30 min at 60 °C. The reaction was reduced *in vacuo* and used as was in the following steps assuming quantitative conversion.

Following Standard Procedure A for solid-phase peptide synthesis and starting with 100 mg Rink amide resin, the following amounts of amino acids were reacted for the times below:

Amino acid	Mass /mg	Coupling time 1 /min	Coupling time 2 /min
**Fmoc-L-Phe-OH**	143	100	-
**Fmoc-L-Thr(^{t}Bu)-OH**	147	120	90
**Fmoc-L-Gly-OH**	110	120	120

Amino acid amounts and reaction times for solid-phase peptide synthesis.

Following the final coupling capping was conducted with *N*^{6}-(*tert*-butoxycarbonyl)-*N*^{2}-(4-(pyridin-3-yl)benzyl)-L-lysine (600 mg, 0.37 mmol), PyCLOCK (205 mg, 0.37 mmol) and DIPEA (129 μL, 0.74 mmol) in DMF (7 mL) for 210 min. Negative Kaiser test suggested capping was complete, and the resin was cleaved and purified by HPLC using the conditions described in Procedure A. Lyophilisation of product containing fractions gave the *peptide* **12** as a white fluffy solid (45 mg, 80% yield).

HPLC elution 6 min, purity >95%; $v_{max.}$ 3600 – 3150 cm^{-1} (N-H), 1640 cm^{-1} (C=O); ^{1}H NMR (500 MHz, CD_3OD) δ 8.98 (1H, s), 8.68 (1H, s), 8.48 – 8.42 (1H, m),

7.85 – 7.76 (3H, m), 7.67 (2H, d, *J* 10.3 Hz), 7.26 – 7.12 (5H, m), 4.59 – 4.52 (1H, m), 4.46 – 4.41 (1H, m), 4.29 – 3.93 (6H, m), 3.17 – 3.09 (1H, m), 2.97 – 2.87 (3H, m), 2.02 – 1.89 (2H, m), 1.75 – 1.62 (2H, m), 1.60 – 1.45 (2H, m), 1.19 – 1.05 (3H, m); ^{13}C NMR (125 MHz, CD_3OD) δ 176.0, 172.8, 172.1, 171.9, 171.6, 169.6, 145.9, 145.2, 140.5, 138.4, 133.1, 132.5, 130.3, 129.5, 129.0, 127.8, 127.3, 61.2, 60.3, 59.9, 50.9, 43.5, 40.2, 38.6, 31.0, 28.0, 22.6, 19.9; LCMS $[M+H]^+$ 618.7, $[M-H]^-$ 616.8, R_t = 2.7; HRMS calc. for $C_{33}H_{44}N_7O_5$ MH^+: 618.3404. Found MH^+: 618.3411.

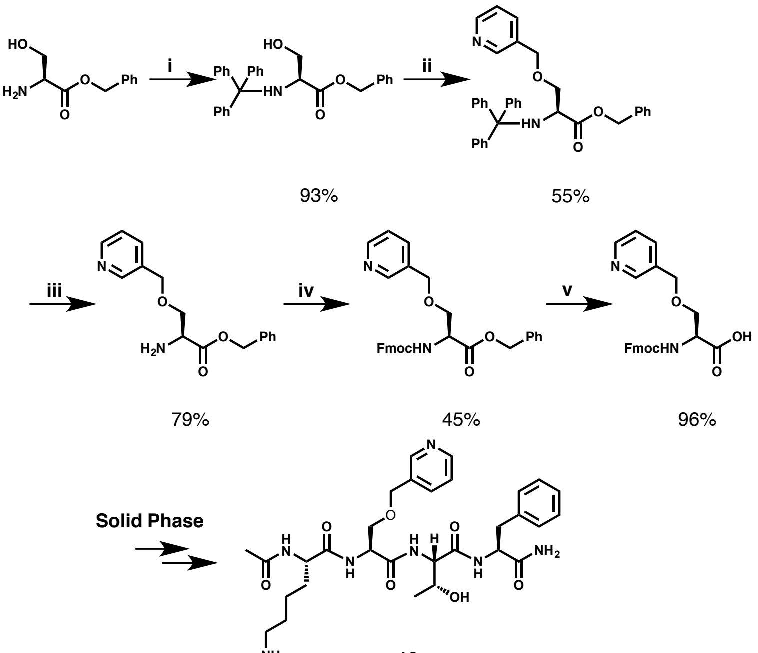

13
57%

Benzyl trityl-L-serinate [6]

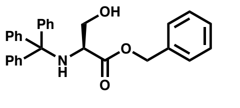

H-L-Ser-OBn (2 g, 8.62 mmol) and triethylamine (2.4 mL, 17.24 mmol) were combined in chloroform (14.0 mL) and cooled to 4 °C. To this was dropwise added a solution of trityl chloride (2.4 g, 8.62 mmol) in chloroform (9.0 mL). The solution was left stirring at 4 °C overnight. The reaction mixture was washed with 10% citric acid (w/v, 25 mL) before the organic layer was dried over anhydrous sodium sulphate, filtered and reduced in vacuo to give the protected serinate [7] as a white amorphous solid (3.5 g, 93% yield).

^{1}H NMR (400 MHz, $CDCl_3$) δ 7.51 – 7.47 (5H, m), 7.33 – 7.18 (15H, m), 4.77 (1H, d, *J* 12.3 Hz), 4.62 (1H, d, *J* 12.3 Hz), 3.74 – 3.69 (1H, m), 3.61 – 3.52 (2H, m); ^{13}C NMR (100 MHz, $CDCl_3$) δ 173.4, 145.6, 135.3, 128.8, 128.7, 128.4, 128.1, 126.7, 71.0, 66.9, 64.9, 57.9; LCMS $[M+Na]^+$ 460.5, R_t = 5.3 min. Data were consistent with those previously reported by Nakajima *et al.* [7].

Benzyl O-(pyridin-3-ylmethyl)-N-trityl-L-serinate [6]

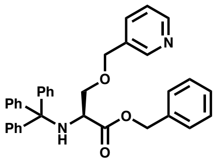

Benzyl trityl-L-serinate (890 mg, 2.03 mmol), 3-(bromomethyl)pyridine hydrobromide (514 mg, 2.03 mmol) and tetrabutylammonium bromide (654 mg, 2.03 mmol) were combined in a solution of toluene (7 mL) and 40% NaOH (2 mL) before being stirred at room temperature for 36 hr. Water (14 mL) was added to the reaction mixture before extracting with EtOAc (3× 20 mL). The combined organics were dried over anhydrous sodium sulphate, filtered and reduced *in vacuo.* The crude product was purified using column chromatography (Biotage, 15 – 100% EtOAc/petroleum ether, v/v) to give the *pyridine serinate* as a white amorphous solid (586 mg, 55% yield).

R_F (10% MeOH/DCM v/v) = 0.4; $\nu_{max.}$ 1741 cm^{-1} (C=O), 1169 cm^{-1} (C-N/C-O), 1033 cm^{-1} (C-N/C-O); ^{1}H NMR (400 MHz, $CDCl_3$) δ 8.56 (1H, dd, *J* 1.5, 4.8 Hz), 8.51 (1H, d *J* 1.6 Hz), 7.60 – 7.52 (7H, m), 7.41 (1H, d, *J* 4.5 Hz), 7.32 – 7.17 (4H, m), 4.79 (1H, d, *J* 12.3 Hz), 4.54 – 4.44 (3H, m), 3.82 (1H, dd, *J* 4.8, 9.2 Hz), 3.73 – 3.65 (1H, m), 3.57 (1H, dd, *J* 6.8, 9.2 Hz), 2.87 (1H, br. d, *J* 9.2 Hz); ^{13}C NMR (100 MHz, $CDCl_3$) δ 173.4, 148.8, 148.6, 145.8, 135.5, 135.4, 133.5, 128.8, 128.6, 128.4, 128.2, 127.9, 126.6, 123.5, 73.0, 71.0, 70.5, 66.7, 56.4; LCMS $[M+H]^+$ 529.6, R_t = 5.3 min; HRMS calc. for $C_{35}H_{33}N_2O_3$ MH^+: 529.2491. Found MH^+: 529.2506.

Benzyl O-(pyridin-3-ylmethyl)-L-serinate

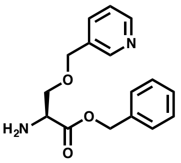

Benzyl O-(pyridin-3-ylmethyl)-N-trityl-L-serinate (100 mg, 0.19 mmol) was dissolved in 20% TFA in DCM (v/v, 4 mL) and stirred at room temperature for 2 hr. The reaction mixture was reduced *in vacuo* to give crude product as the TFA salt, which was dissolved in water (4 mL) and extracted with EtOAc (3× 5 mL) before basifying the aqueous to pH 9 and extracting with EtOAc again (3× 5 mL). The basic extracts were dried over anhydrous sodium sulphate, filtered and reduced *in vacuo* to give the *pyridine serinate* as a colourless oil (44 mg, 79% yield).

R_F (10% MeOH/DCM v/v) = 0.3; $\nu_{max.}$ 3333 cm^{-1} (N-H), 1720 cm^{-1} (C=O), 1660 cm^{-1} (N-H), 1203 cm^{-1} (C-O ester), 1103 cm^{-1} (C-O ether), 1019 cm^{-1} (N-H); ^{1}H NMR (400 MHz, $CDCl_3$) δ 8.50 (1H, dd, *J* 1.6, 4.8 Hz), 8.48 (1H, d, *J* 1.6 Hz), 7.54 (1H, ddd, *J* 1.7, 2.2, 7.8 Hz), 7.31 (5H, br. s), 7.21 (1H, ddd, *J* 0.7, 4.8, 7.8 Hz), 5.19 (1H, d, *J* 12.2 Hz), 5.13 (1H, d, *J* 12.2 Hz), 4.51 (1H, d, *J* 12.2 Hz), 4.45 (1H, d, *J* 12.2 Hz), 3.81 – 3.76 (1H, m), 3.74 – 3.65 (2H, m), 2.01 (2H, br. s); ^{13}C NMR (100 MHz, $CDCl_3$) δ 173.6, 149.2, 149.0, 135.6, 135.3, 133.2, 128.6, 128.4, 128.2, 123.4, 72.3, 70.8, 66.9, 54.9; LCMS [M+H]$^+$ 287.2, R_t = 0.4 min; HRMS calc. for $C_{16}H_{19}N_2O_3$ MH$^+$: 287.1396. Found MH$^+$: 287.1425.

Benzyl N-(((9H-fluoren-9-yl)methoxy)carbonyl)-O-(pyridin-3-ylmethyl)-L-serinate

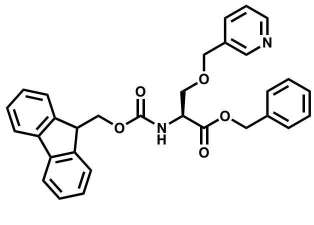

To a solution of benzyl O-(pyridin-3-ylmethyl)-L-serinate (131 mg, 0.46 mmol) in THF (5.0 mL) was added N-(9-Fluorenylmethoxycarbonyloxy)succinimide (163 mg,

0.48 mmol) and sat. sodium bicarbonate (5.0 mL) before stirring at room temperature for 18 hr. The reaction mixture was reduced *in vacuo* before the residue was diluted with water (15 mL), neutralised and extracted with EtOAc (3× 15 mL). The combined organics were washed with brine (15 mL), dried over anhydrous sodium sulphate, filtered and reduced *in vacuo*. The crude product was purified by column chromatography (Biotage, 20 – 100% EtOAc/petroleum ether v/v). Product containing fractions were reduced *in vacuo* to give the *pyridine serinate* as a colourless solid (32 mg, 45% yield).

R_F (80% MeOH/DCM v/v) = 0.6; $\nu_{max.}$ 1714 cm^{-1} (C=O), 1245 cm^{-1} (C-O), 1194 cm^{-1} (C-O), 1033 cm^{-1} (N-H); ^{1}H NMR (400 MHz, $CDCl_3$) δ 8.55 (1H, br. s), 8.49 (1H, br. s), 7.76 (2H, d, *J* 6.0 Hz), 7.60 (2H, dd, *J* 1.6, 6.3 Hz), 7.51 (1H, d, *J* 6.1 Hz), 7.40 (2H, t, *J* 6.0 Hz), 7.34 – 7.28 (7H, m), 7.22 (1H, dd, *J* 3.9, 5.9 Hz), 5.78 (1H, d, *J* 6.9 Hz), 5.26 (1H, d, *J* 9.8 Hz), 5.17 (1H, d, *J* 9.8 Hz), 4.61 (1H, dt, *J* 2.4, 6.8 Hz), 4.53 – 4.34 (4H, m), 4.23 (1H, t, *J* 5.7 Hz), 3.95 (1H, dd, *J* 2.4, 7.5 Hz), 3.77 (1H, dd, *J* 2.5, 7.6 Hz); ^{13}C NMR (100 MHz, $CDCl_3$) δ 170.0, 156.0, 149.3, 148.9, 143.9, 143.7, 141.3, 135.3, 132.9, 128.6, 128.5, 128.3, 127.7, 127.1, 125.1, 123.5, 120.0, 70.8, 70.4, 67.4, 67.2, 54.5, 47.1; LCMS [M+H]$^+$ 509.4, R_t = 2.0 min; HRMS calc. for $C_{31}H_{29}N_2O_5$ MH$^+$: 509.2076. Found MH$^+$: 509.2094.

N-(((9H-Fluoren-9-yl)methoxy)carbonyl)-O-(pyridin-3-ylmethyl)-L-serine

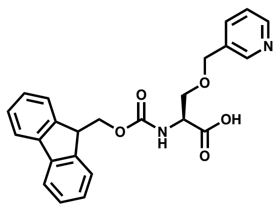

A solution of benzyl N-(((9H-fluoren-9-yl)methoxy)carbonyl)-O-(pyridin-3-ylmethyl)-L-serinate (165 mg, 0.32 mmol) in methanol (12 mL) was purged with nitrogen before the addition of 10% palladium hydroxide on carbon (41 mg, 25 w%) and purging with hydrogen. The reaction was then sealed and stirred under an atmosphere of

hydrogen for 2 hr. The reaction mixture was filtered through Celite® and concentrated *in vacuo* to give the *pyridine serine* as a white solid (128 mg, 96% yield).

R_F (20% MeOH/DCM v/v) = 0.2; $\nu_{max.}$ 2937 cm^{-1} (O-H, COOH), 1760 cm^{-1} (C=O), 1424 cm^{-1} (O-H), 1053 cm^{-1} (C-N/C-O), 1033 cm^{-1} (C-N/C-O); ^{1}H NMR (400 MHz, CD_3OD) δ 8.51 (1H, s), 8.43 (1H, s), 7.83 – 7.71 (3H, m), 7.65 (2H, dd, *J* 4.2, 7.0 Hz), 7.40 – 7.32 (3H, m), 7.30 – 7.23 (2H, m), 4.57 (2H, q, *J* 11.1 Hz), 4.44 – 4.27 (3H, m), 4.19 (1H, t, *J* 6.8 Hz), 3.90 (1H, dd, *J* 5.3, 9.5 Hz), 3.83 (1H, dd, *J* 3.3, 9.5 Hz); ^{13}C NMR (100 MHz, CD_3OD) δ 176.3, 158.4, 149.2, 149.1, 145.2, 142.5, 137.7, 136.1, 128.8, 128.1, 126.2, 125.1, 120.9, 72.1, 71.2, 67.9, 57.0, 48.3; LCMS $[M+H]^+$ 419.5, $[M-H]^-$ 417.6, R_t = 3.8 min; HRMS calc. for $C_{24}H_{23}N_2O_5$ MH^+: 419.1607. Found MH^+: 419.1610.

(S)-2-Acetamido-6-amino-N-((S)-1-(((2S,3R)-1-(((S)-1-amino-1-oxo-3-phenylpropan-2-yl)amino)-3-hydroxy-1-oxobutan-2-yl)amino)-1-oxo-3-(pyridin-3-ylmethoxy)propan-2-yl)hexanamide ***13***

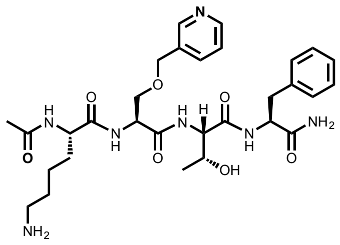

Following Standard Procedure A for solid-phase peptide synthesis and starting with 100 mg Rink amide resin, the following amounts of amino acids were reacted for the times below:

Amino acid	Mass /mg	Coupling agent	Coupling time /min
Fmoc-L-Phe-OH	143	PyBop	100
Fmoc-L-Thr(^{t}Bu)-OH	147	PyBop	100
N-(((9*H*-fluoren-9-yl)methoxy)carbonyl)-*O*-(pyridin-3-ylmethyl)-L-serine	155	PyClock	600
Fmoc-L-Lys(Boc)-OH	174	PyBop	80

Amino acid amounts, coupling agents and reaction times.

Following the final coupling, capping was conducted with acetic anhydride (35 μL, 0.37 mmol) in DMF (7 mL) for 20 min. Negative Kaiser test suggested capping was complete and the resin was cleaved and purified by HPLC using the conditions described in Procedure A. Lyophilisation of product containing fractions gave the *peptide* **13** as a white fluffy solid (26 mg, 57% yield).

HPLC elution 12 min, purity >95%; $\nu_{max.}$ 3301 cm^{-1} (O-H), 1667 cm^{-1} (C=O), 1203 cm^{-1} (C-N/C-O), 1134 cm^{-1} (C-N/C-O); ^1H NMR (500 MHz, CD_3OD) δ 8.88 – 8.66 (2H, m), 8.41 (1H, dd, *J* 7.9, 20.9 Hz), 7.95 (1H, dd, *J* 7.8, 13.5 Hz), 7.31 – 7.11 (5H, m), 4.81 – 4.56 (4H, m), 4.39 – 4.11 (3H, m), 3.92 (1H, dd, *J* 5.5, 9.6 Hz), 3.85 (1H, dd, *J* 4.8, 9.7 Hz), 3.25 – 3.14 (1H, m), 3.02 – 2.88 (3H, m), 2.00 (3H, s), 1.90 – 1.64 (4H, m), 1.59 – 1.40 (2H, m), 1.10 (3H, dd, *J* 6.4, 14.5 Hz); ^{13}C NMR (125 MHz, CD_3OD) δ 175.9, 174.6, 173.6, 172.0, 171.9, 144.6, 143.3, 143.2, 139.6, 138.5, 130.4, 129.4, 127.8, 127.7, 71.2, 70.2, 68.4, 60.1, 56.0, 55.6, 54.9, 40.5, 38.7, 32.3, 28.2, 23.7, 22.4, 20.0; LCMS $[M+H]^+$ 614.5, $[M-H]^-$ 612.6, R_t = 0.4 min; HRMS calc. for $C_{30}H_{44}N_7O_7$ MH^+: 614.3302. Found MH^+: 614.3302.

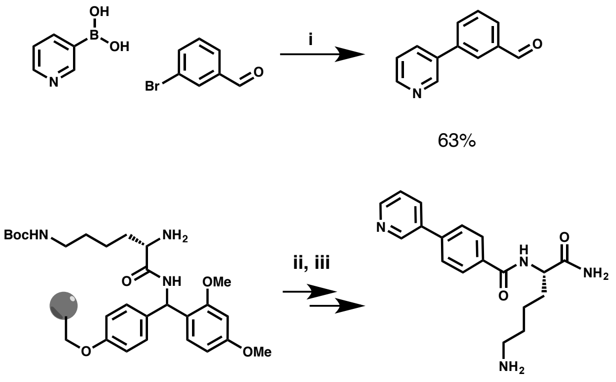

14
43%

i 0.04 eq Pd(dppf)Cl_2, 2 eq K_2CO_3, DME:water (3:1), 2 hr, 120 ºC.
ii 5 eq 4-(pyridin-3-yl)benzoic acid, 5 eq PyBop, 10 eq DIPEA, DMF, 2x 2 hr.
iii TFA: H_2O 95: 5, 4 hr.

3-(Pyridin-3-yl)benzaldehyde

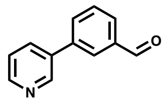

Following Standard Procedure B for Suzuki couplings, 3-bromobenzaldehyde (300 mg, 1.62 mmol) and 3-pyridineboronic acid (200 mg, 1.62 mmol) were combined and reacted as described above. The crude product was purified by column chromatography (Biotage, eluent 20 – 100% EtOAc in petroleum ether v/v) to give the pyridine aldehyde [8] as a yellow oil (189 mg, 63% yield).

R_F (2:1 EtOAc in petroleum ether v/v) = 0.3. ^{1}H NMR (400 MHz, $CDCl_3$) δ 10.11 (1H, s), 8.91 (1H, dd, *J* 0.7, 2.3 Hz), 8.66 (1H, dd, *J* 1.6, 4.9 Hz), 8.10 (1H, t, *J* 1.6 Hz), 7.98 (1H, ddd, *J* 1.7, 2.3, 7.9 Hz), 7.94 (1H dt, *J* 1.3, 7.6 Hz), 7.87 (1H, ddd, *J* 1.2, 1.9, 7.7 Hz), 7.68 (1H, t, *J* 7.7 Hz), 7.46 (1H, ddd, *J* 0.8, 4.9, 7.9 Hz); ^{13}C NMR (100 MHz, $CDCl_3$) δ 191.9, 148.6, 147.7, 138.6, 137.2, 135.6, 135.0, 133.0, 130.0, 129.7, 128.0, 124.0; LCMS $[M+H]^+$ 184.2, R_t = 3.1 min. HRMS calc. for $C_{12}H_{10}NO$ MH^+: 184.0762. Found MH^+: 184.0781. Data were consistent with those previously reported by Wang *et al.* [8].

(S)-N-(1,6-Diamino-1-oxohexan-2-yl)-4-(pyridin-3-yl)benzamide ***14***

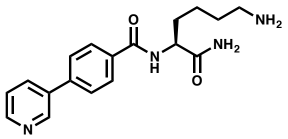

Following Standard Procedure A for solid-phase peptide synthesis and starting with 260 mg of Rink amide resin, a double coupling was performed with 2× 415 mg of Fmoc-L-Lys(Boc)-OH for 2× 60 min. After deprotecting, the lysine was then capped using PyBOP and 4-(pyridine-3-yl)benzoic acid (2× 106 mg, 2× 130 min). After cleavage of the peptide from the resin and purification by HPLC the *pyridine lysine amide* **14** was obtained as a white powder (25 mg, 43% yield).

$\nu_{max.}$ 3395 cm^{-1} (N-H), 1672 cm^{-1} (C=O); ^{1}H NMR (500 MHz, D_2O) δ 8.97 (1H, d, *J* 2.0 Hz), 8.74 – 8.76 (1H, m), 8.65 (1H, d, *J* 5.7 Hz), 8.03 – 8.01 (1H, m), 7.83 – 7.82 (2H, m), 7.74 – 7.72 (2H, m), 4.39 – 4.34 (1H, m), 2.87 (2H, t, *J* 7.6 Hz), 1.88 – 1.71 (2H, m), 1.64 – 1.53 (2H, m), 1.48 – 1.32 (2H, m); ^{13}C NMR (125 MHz, D_2O) δ 176.7, 170.2, 145.0, 139.8, 139.4, 139.3, 137.0, 134.2, 128.4, 127.7, 127.4, 54.1, 39.1, 30.4, 26.2, 22.2; LCMS $[M+H]^+$ 327.1, $[M-H]^-$ 325.3, R_t = 2.3 min; HRMS calc. for $C_{18}H_{23}N_4O_2$ MH^+: 327.1821. Found MH^+: 327.1834.

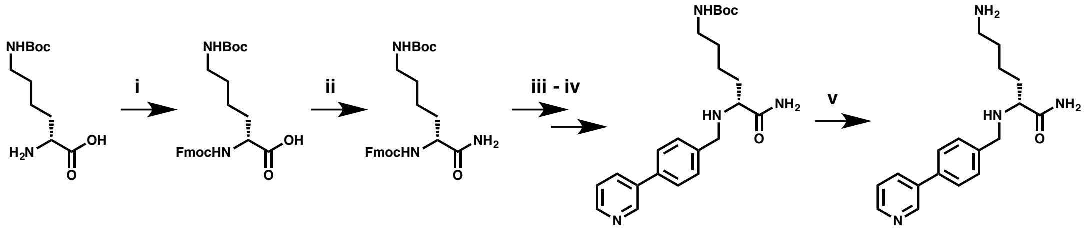

15

97% 56% 27% Quantitative

i 1.05 ee FmocOSu, THF: sat. $NaHCO_3$ (1:1), 18 hr.
ii 2 eq NH_4OH 35% solution, 1 eq HOBt, 1.15 eq EDC, THF, 18 hr.
iii 20% Piperidine in DMF, 18 hr.
iv 1 eq 4-(3-pyridinyl)benzaldehyde, 1.7 eq $NaBH(OAc)_3$, Acetic acid 1% bv, THF, 33 hr.
v 4 M HCl in dioxane, 4 hr.

N^2-(((9H-Fluoren-9-yl)methoxy)carbonyl)-N^6-(tert-butoxycarbonyl)-D-lysine

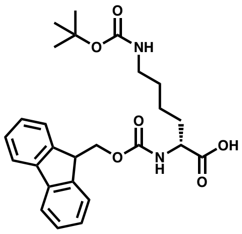

To a solution of H-D-Lys(Boc)-OH (300 mg, 1.22 mmol) in THF (10 mL) was added *N*-(9-Fluorenylmethoxycarbonyloxy)succinimide (432 mg, 1.28 mmol) and sat. sodium bicarbonate (10 mL) before stirring at room temperature for 18 hr. The reaction mixture was reduced *in vacuo* before the residue was diluted with water (5 mL) and extracted with EtOAc (3× 15 mL). The combined organics were dried over anhydrous sodium sulphate, filtered and reduced *in vacuo* to give crude product, which was purified by column chromatography (Biotage, 0 – 20% MeOH/DCM v/v). Product containing fractions were reduced *in vacuo* to give the Fmoc-D-lysine [9] as a white solid (555 mg, 97% yield).

R_F (10% MeOH/DCM v/v) = 0.5; ^{1}H NMR (400 MHz, CD_3OD) δ 7.81 (2H, d, *J* 7.5 Hz), 7.69 (2H, t, *J* 7.0 Hz), 7.40 (2H, t, *J* 7.4 Hz), 7.32 (2H, td, *J* 1.1, 7.4 Hz), 4.36 (2H, dd, *J* 2.5, 6.9 Hz), 4.24 (1H, t, *J* 6.9 Hz), 4.12 (1H, dd, *J* 4.5, 9.0 Hz), 3.05 (2H, t, *J* 6.7 Hz), 1.91 – 1.80 (1H, m), 1.76 – 1.64 (1H, m), 1.55 – 1.35 (13H, m); ^{13}C NMR (100 MHz, CD_3OD) δ 158.7, 145.4, 145.2, 142.6, 142.5, 128.8, 128.2, 126.3, 120.9, 79.9, 67.9, 55.6, 48.4, 41.1, 32.5, 30.5, 28.8, 24.2; LCMS $[M+H]^+$ 469.5, $[M-H]^-$ 467.7,

R_t = 4.4 min; HRMS calc. for $C_{26}H_{33}N_2O_6$ MH^+: 469.2386. Found MH^+: 469.2369. Data were consistent with those previously reported by Watts *et al.* [9].

(9H-Fluoren-9-yl)methyl tert-butyl (6-amino-6-oxohexane-1,5-diyl)(R)-dicarbamate

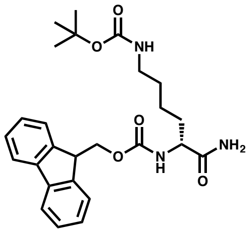

N^2-(((9H-Fluoren-9-yl)methoxy)carbonyl)-N^6-(tert-butoxycarbonyl)-D-lysine (500 mg, 1.07 mmol), hydroxybenzotriazole (144 mg, 1.07 mmol) and 1-ethyl-3-(3-dimethylaminopropyl)carbodiimide (236 mg, 1.23 mmol) were dissolved in THF (18 mL) and stirred at room temperature for 30 min before the addition of 35% ammonium hydroxide (47 μL, 2.14 mmol). The reaction was stirred for a further 18 hr. The mixture was then concentrated *in vacuo* before diluting with EtOAc (20 mL) and washing with sat. sodium bicarbonate (20 mL) and brine (20 mL). The combined organics were dried over anhydrous magnesium sulphate, filtered and reduced *in vacuo* to give the crude product, which was purified by column chromatography (Biotage, 20 – 100% EtOAc/petroleum ether v/v). Product fractions were reduced *in vacuo* to give the *Fmoc-D-lysine amide* as a white solid (281 mg, 56% yield).

R_F (60% EtOAc/petroleum ether v/v) = 0.1; $v_{max.}$ 3321 cm^{-1} (N-H), 1660 cm^{-1} (C=O), 1249 cm^{-1} (C-O), 1033 cm^{-1} (C-N); ^1H NMR (500 MHz, $CDCl_3$) δ 7.76 (2H, d, *J* 7.6 Hz), 7.59 (2H, d, *J* 7.3 Hz), 7.40 (2H, t, *J* 7.4 Hz), 7.31 (2H, td, *J* 0.8, 7.4 Hz), 6.14 (1H, br. s), 5.56 (2H, br. d, *J* 16.6 Hz), 4.63 (1H, br. s), 4.48 – 4.36 (2H, m), 4.23 – 4.13 (2H, m), 3.16 – 3.04 (2H, m), 1.93 – 1.61 (2H, m), 1.56 – 1.34 (13H, m); ^{13}C NMR (125 MHz, $CDCl_3$) δ 174.0, 156.3, 143.8, 143.7, 141.3, 127.7, 127.1, 125.0, 120.0, 79.3, 66.9, 54.3, 47.2, 39.7, 31.7, 29.6, 28.4, 22.3; LCMS $[M+H]^+$ 468.7, R_t = 4.3 min; HRMS calc. for $C_{26}H_{34}N_3O_5$ MH^+: 468.2498. Found MH^+: 468.2502.

Tert-Butyl (R)-(6-amino-6-oxo-5-((4-(pyridin-3-yl)benzyl)amino)hexyl)carbamate [10]

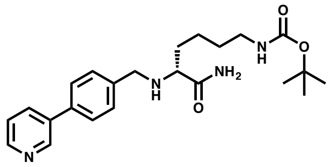

(9H-Fluoren-9-yl)methyl tert-butyl (6-amino-6-oxohexane-1,5-diyl)(*R*)-dicarbamate (270 mg, 0.58 mmol) was dissolved in 20% piperidine in DMF (v/v, 3 mL) and left stirring for 18 hr. The solvent was removed *in vacuo* to give the crude deprotected amino acid. This was combined with 4-(3-pyridinyl)benzaldehyde (106 mg, 0.58 mmol) and acetic acid (40 µL, 1% v/v) in THF (3.96 mL) before the addition of triacetoxyborohydride (135 mg, 0.64 mmol) and stirring for 18 hr. After this time, more triacetoxyborohydride (67 mg, 0.32 mmol) was added due to unreacted starting materials and the reaction stirred for a further 15 hr. The reaction was quenched with sat. potassium carbonate (6 mL) for 1 hr before extraction with EtOAc (3× 6 mL). The combined organics were washed with brine (6 mL), dried over anhydrous magnesium sulphate, filtered and reduced *in vacuo* to give the crude product. Further purification affected by column chromatography (Biotage, eluent 0 – 20% MeOH/DCM v/v) gave the *pyridine lysine amide* as a yellow solid (65 mg, 27% yield).

R_F (10% MeOH/DCM v/v) = 0.3; $\nu_{max.}$ 3326 cm^{-1} (N-H), 1674 cm^{-1} (C=O), 1169 cm^{-1} (C-O), 1033 cm^{-1} (C-N); ^1H NMR (400 MHz, $CDCl_3$) δ 8.81 (1H, d, *J* 2.0 Hz), 8.56 (1H, dd, *J* 1.4, 4.8 Hz), 7.85 (1H, dt, *J* 1.9, 7.9 Hz), 7.53 (2H, d, *J* 8.1 Hz), 7.41 (2H, d, *J* 8.1 Hz), 7.35 (1H, dd, *J* 4.8, 7.9 Hz), 7.11 (1H, d, *J* 4.0 Hz), 6.25 (1H, br. s), 4.79 (1H, br. s), 3.86 (1H, d, *J* 13.4 Hz), 3.73 (2H, d, *J* 13.4 Hz), 3.15 (1H, dd, *J* 5.6, 7.0 Hz), 3.12 – 3.03 (2H, m), 1.81 – 1.58 (2H, m), 1.51 – 1.34 (13H, m); ^{13}C NMR (100 MHz, $CDCl_3$) δ 177.5, 156.1, 148.4, 148.1, 139.6, 136.7, 136.3, 134.3, 128.8, 127.3, 123.6, 79.1, 62.1, 52.2, 40.0, 33.2, 29.9, 28.4, 22.9; LCMS $[M+H]^+$ 413.6, R_t = 3.1 min; HRMS calc. for $C_{23}H_{33}N_4O_3$ MH^+: 413.2553. Found MH^+: 413.2575.

(R)-6-Amino-2-((4-(pyridin-3-yl)benzyl)amino)hexanamide ***15***

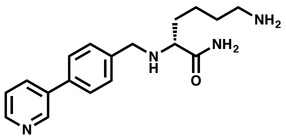

Tert-Butyl (*R*)-(6-amino-6-oxo-5-((4-(pyridin-3-yl)benzyl)amino)hexyl)carbamate (60 mg, 0.15 mmol) was dissolved in 4 M HCl in dioxane (4 mL) and stirred for 3 hr at room temperature. The reaction was reduced *in vacuo* to give the crude compound, which was left on the high vacuum line overnight and used without further purification. The *pyridine lysine amide* **15** was isolated as the HCl salt, a yellow amorphous solid (65 mg, quantitative yield).

$\nu_{max.}$ 3389 cm^{-1} (N-H), 1687 cm^{-1} (C=O), 1033 cm^{-1} (C-N); ^{1}H NMR (400 MHz, CD_3OD) δ 9.29 (1H, s), 9.01 (1H, d, *J* 7.5 Hz), 8.93 (1H, d, *J* 5.0 Hz), 8.25 (1H, t, *J* 6.0 Hz), 7.97 (2H, d, *J* 7.5 Hz), 7.82 (2H, d, *J* 7.4 Hz), 4.37 (1H, d, *J* 12.8 Hz), 4.29 (1H, d, *J* 12.9 Hz), 4.06 (1H, t, *J* 5.6 Hz), 2.99 (2H, t, *J* 7.1 Hz), 2.12 – 1.96 (2H, m), 1.84 – 1.70 (2H, m), 1.63 – 1.52 (2H, m); ^{13}C NMR (100 MHz, CD_3OD) δ 170.9, 146.0, 141.4, 141.0, 140.9, 136.0, 134.2, 132.8, 129.3, 129.0, 61.0, 50.8, 40.3, 31.0, 28.0, 22.9; HRMS calc. for $C_{18}H_{25}N_4O$ MH^+: 313.2028. Found MH^+: 313.2044; $[\alpha]_D^{25}$ = -9.4.

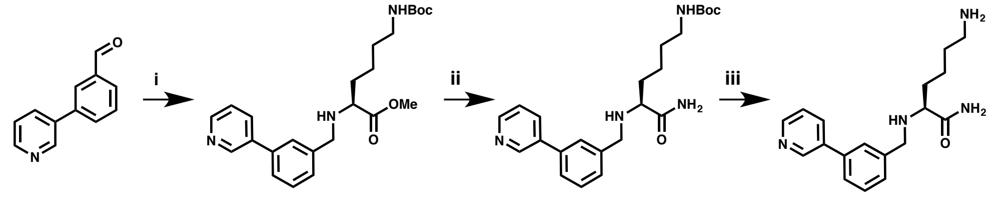

16

69% 24% Quantitative

i 1 eq H-Lys(Boc)-OMe.HCl, 1.1 eq $NaBH(OAc)_3$, THF, 18 hr.
ii 5 eq Mg_3N_2, MeOH, 80 °C, 24 hr.
iii 4 M HCl in dioxane, 3 hr.

Methyl N⁶-(tert-butoxycarbonyl)-N²-(3-(pyridin-3-yl)benzyl)-L-lysinate [4]

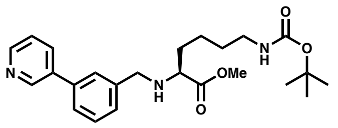

H-Lys(Boc)-OMe.HCl (310 mg, 1.05 mmol) and 3-(pyridine-3-yl)benzaldehyde (197 mg, 1.08 mmol) were combined in THF (6 mL) before addition of sodium triacetoxyborohydride (244 mg, 1.16 mmol) and stirring at room temperature for 18 hr. The reaction was quenched with sat. potassium carbonate (10 mL) for 1 hr before extraction with EtOAc (3× 15 mL). The combined organics were washed with brine (15 mL), dried over anhydrous magnesium sulphate, filtered and reduced in vacuo to give the crude product. Further purification by column chromatography (Biotage, eluent 0 – 20% MeOH/DCM v/v) gave contaminated with aldehyde starting material. The crude product was dissolved in EtOAc (15 mL) and washed with sat. sodium bisulphite (15 mL) before the organic layer was dried over magnesium sulphate, filtered and reduced in vacuo to give the *pryridine methyl lysinate* as a pale yellow oil (311 mg, 69% yield).

R_F (10% MeOH in DCM v/v) = 0.5. $\nu_{max.}$ 3330 cm^{-1} (N-H secondary amine), 1740 cm^{-1} (C=O), 1690 cm^{-1} (C=O), 1167 cm^{-1} (C-O), 1025 cm^{-1} (C-N); ^1H NMR (500 MHz, $CDCl_3$) δ 8.84 (1H, dd, *J* 0.6, 2.4 Hz), 8.59 (1H, dd, *J* 1.6, 4.8 Hz), 7.88 (1H, ddd, *J* 1.7, 2.3, 7.9 Hz), 7.55 (1H, br. s), 7.49 – 7.41 (2H, m), 7.39 – 7.34 (2H, m), 4.53 (1H, br. s), 4.32 (1H, br. s), 3.89 (1H, d, *J* 13.2 Hz), 3.78 – 3.67 (4H, m), 3.29 (1H, t, *J* 6.7 Hz), 3.09 (2H, d, *J* 5.8 Hz), 1.80 – 1.60 (2H, m), 1.48 – 1.35 (13H, m);

^{13}C NMR (125 MHz, $CDCl_3$) δ 175.8, 155.9, 148.5, 148.3, 140.8, 137.9, 136.6, 134.4, 129.2, 128.0, 127.0, 125.9, 123.5, 79.1, 60.6, 52.0, 51.8, 40.3, 33.2, 29.8, 28.4, 23.0; LCMS $[M+H]^+$ 428.6, R_t = 3.4 min; HRMS calc. for $C_{24}H_{34}N_3O_4$ MH^+: 428.2549. Found MH^+: 428.2579.

Tert-butyl (S)-(6-amino-6-oxo-5-((3-(pyridin-3-yl)benzyl)amino)hexyl)carbamate [5]

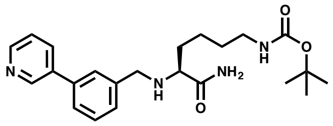

A solution of methyl N^6-(tert-butoxycarbonyl)-N^2-(3-(pyridin-3-yl)benzyl)-L-lysinate (300 mg, 0.70 mmol) was made up in a 10 – 20 mL microwave vial (Biotage) with methanol (5 mL) before cooling to 0 °C, whereupon magnesium nitride (355 mg, 3.50 mmol) was added in one portion. The vial was sealed and allowed to warm to room temperature in a water bath for 1 hr. The reaction was then heated to 80 °C for 24 hr, carefully monitoring the state of the cap over the first 3 hr. CAUTION: these reactions must be carried out behind a blast shield. The reaction was cooled to room temperature and vented carefully before diluting with chloroform (25 mL) and water (25 mL). The reaction was neutralised with HCl 3 N before the layers were separated and the aqueous layer further extracted with chloroform (3× 25 mL). The combined organics were dried over anhydrous magnesium sulphate, filtered and reduced *in vacuo*. The crude product was purified by column chromatography (Biotage, eluent 0 – 20% MeOH/DCM v/v) to give the *pyridine lysine amide* as a yellow solid (68 mg, 24% yield).

R_F (10% MeOH in DCM v/v) = 0.3. $\nu_{max.}$ 3324 cm^{-1} (N-H secondary amine), 1669 cm^{-1} (C=O), 1168 cm^{-1} (C-O), 1033 cm^{-1} (C-N); ^1H NMR (400 MHz, $CDCl_3$) δ 8.85 (1H, d, *J* 1.8 Hz), 8.61 (1H, dd, *J* 1.6, 4.8 Hz), 7.90 (1H, ddd, *J* 1.7, 2.3, 7.9 Hz), 7.55 – 7.35 (5H, m), 7.08 (1H, d, *J* 3.4 Hz), 5.82 (1H, br. s), 4.66 (1H, t, *J* 5.7 Hz), 3.91 (1H, d, *J* 13.3 Hz), 3.79 (1H, d, *J* 13.3 Hz), 3.19 (1H, dd, *J* 5.5, 7.1 Hz),

3.15 – 3.06 (2H, m), 1.83 – 1.62 (2H, m), 1.53 – 1.38 (13H, m); ^{13}C NMR (100 MHz, $CDCl_3$) δ 177.3, 156.1, 148.5, 148.3, 140.5, 138.2, 136.5, 134.5, 129.4, 127.9, 126.9, 126.2, 123.6, 79.1, 62.2, 52.7, 40.0, 33.1, 29.9, 28.4, 22.9; LCMS $[M+H]^+$ 413.3, R_t = 1.1 min; HRMS calc. for $C_{23}H_{33}N_4O_3$ MH^+: 413.2553. Found MH^+: 413.2573.

(S)-6-Amino-2-((3-(pyridin-3-yl)benzyl)amino)hexanamide **16**

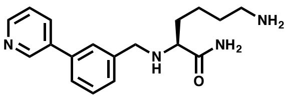

Tert-butyl (*S*)-(6-amino-6-oxo-5-((3-(pyridin-3-yl)benzyl)amino)hexyl)carbamate (51 mg, 0.12 mmol) was dissolved in 4 M HCl in dioxane (4 mL) and stirred for 3 hr at room temperature. The reaction was reduced *in vacuo* to give the crude compound, which was left on the high vacuum line overnight and used without further purification. The *pyridine lysine amide* **16** was isolated as the HCl salt, a yellow amorphous solid (50 mg, quantitative yield).

$\nu_{max.}$ 3370 cm^{-1} (N-H), 1632 cm^{-1} (C=O), 1033 cm^{-1} (C-N); ^{1}H NMR (400 MHz, CD_3OD) δ 9.33 (1H, s), 9.06 (1H, d, *J* 8.0 Hz), 8.92 (1H, d, *J* 5.4 Hz), 8.25 (1H, dd, *J* 5.9, 7.6 Hz), 8.16 (1H, s), 7.96 (1H, d *J* 7.5 Hz), 7.76 (1H, d, *J* 7.5 Hz), 7.70 (1H, t, *J* 7.6 Hz), 4.40 (1H, d, *J* 13.0 Hz), 4.31 (1H, d, *J* 12.9 Hz), 4.08 (1H, t, *J* 6.1 Hz), 2.98 (2H, t, *J* 7.3 Hz), 2.14 – 1.96 (2H, m), 1.83 – 1.69 (2H, m), 1.64 – 1.51 (2H, m); ^{13}C NMR (100 MHz, CD_3OD) δ 170.9, 146.0, 141.4, 141.1, 141.0, 135.5, 133.6, 133.3, 131.5, 131.0, 129.7, 129.0, 61.1, 51.1, 40.2, 31.1, 28.1, 22.9; HRMS calc. for $C_{18}H_{25}N_4O$ MH^+: 313.2028. Found MH^+: 313.2056.

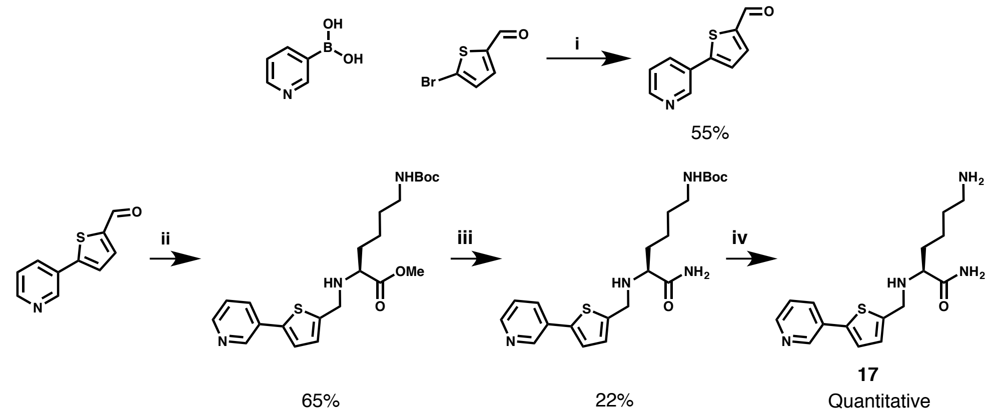

i 0.04 eq Pd(dppf)Cl_2, 2 eq K_2CO_3, DME:water (3:1), 2 hr, 120 °C.
ii 1 eq H-Lys(Boc)-OMe.HCl, 1.1 eq NaBH$(OAc)_3$, THF, 18 hr.
iii 5 eq Mg_3N_2, MeOH, 80 °C, 24 hr.
iv 4 M HCl in dioxane, 3 hr.

5-(Pyridin-3-yl)thiophene-2-carbaldehyde

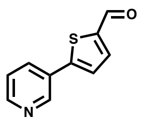

Following Standard Procedure B for Suzuki couplings, 5-bromothiophene-2-carbaldehyde (309 mg, 1.62 mmol) and 3-pyridineboronic acid (200 mg, 1.62 mmol) were combined and reacted as described above. The crude product was purified by column chromatography (Biotage, eluent 20 – 100% EtOAc/petroleum ether v/v) to give the pyridine carbaldehyde [11] as a yellow solid (165 mg, 55% yield).

R_F (30% EtOAc/petroleum ether v/v) = 0.2; ^{1}H NMR (400 MHz, $CDCl_3$) δ 9.94 (1H, s), 8.98 (1H, s), 8.65 (1H, d, *J* 4.1 Hz), 8.02 (1H, d, *J* 8.0 Hz), 7.80 (1H, d, *J* 3.9 Hz), 7.51 – 7.44 (2H, m); ^{13}C NMR (100 MHz, $CDCl_3$) δ 182.7, 149.1, 148.9, 146.1, 144.0, 137.2, 134.7, 129.8, 125.5, 124.3; LCMS $[M+H]^+$ 190.2, R_t = 3.5 min; HRMS calc. for $C_{10}H_8NOS$ MH^+: 190.0327. Found MH^+: 190.0336. Data were consistent with those previously reported by Barder *et al.*[11]

Methyl N^{6}-(tert-butoxycarbonyl)-N^{2}-((5-(pyridin-3-yl)thiophen-2-yl)methyl)-L-lysinate [4]

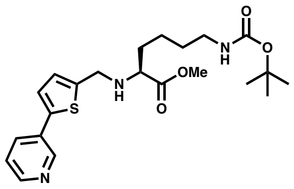

H-Lys(Boc)-OMe.HCl (441 mg, 1.48 mmol) and 5-(pyridine-3-yl)thiophene-2-carbaldehyde (389 mg, 1.53 mmol) were combined in THF (9 mL) before addition of sodium triacetoxyborohydride (344 mg, 1.63 mmol) and stirring at room temperature for 18 hr. The reaction was quenched with sat. potassium carbonate (10 mL) for 1 hr before extraction with EtOAc (3× 15 mL). The combined organics were washed with brine (15 mL), dried over anhydrous magnesium sulphate, filtered and reduced *in vacuo* to give the crude product. Purification by column chromatography (Biotage, eluent 0 – 20% MeOH/DCM v/v) gave contaminated with aldehyde starting material. The crude product was dissolved in EtOAc (15 mL) and washed with sat. sodium bisulphite (15 mL) before the organic layer was dried over magnesium sulphate, filtered and reduced *in vacuo* to give the *pyridine methyl lysinate* as a yellow oil (415 mg, 65% yield).

R_F (10% MeOH in DCM v/v) = 0.5. $v_{max.}$ 3320 cm^{-1} (N-H secondary amine), 1735 cm^{-1} (C=O, ester), 1700 cm^{-1} (C=O, NHBoc); ^{1}H NMR (500 MHz, $CDCl_3$) δ 8.83 (1H, dd, *J* 0.7, 2.4 Hz), 8.48 (1H, dd, *J* 1.6, 4.8 Hz), 7.82 (1H, ddd, *J* 1.6, 2.3, 8.0 Hz), 7.28 (1H, ddd, *J* 0.8, 4.8, 8.0 Hz), 7.18 (1H, d, *J* 3.6 Hz), 6.90 (1H, d, *J* 3.7 Hz), 4.55 (1H, br. s), 4.05 (1H, dd, *J* 0.8, 14.2 Hz), 3.81 (1H, dd, *J* 0.6, 14.1 Hz), 3.73 (3H, s), 3.33 (1H, dd, *J* 6.1, 7.3 Hz), 3.10 (2H, dd, *J* 6.8, 12.6 Hz), 1.73 – 1.57 (2H, m), 1.51 – 1.34 (13H, m); ^{13}C NMR (125 MHz, $CDCl_3$) δ 175.5, 155.9, 148.2, 146.7, 144.9, 139.4, 132.7, 130.6, 126.1, 123.7, 123.6, 79.1, 60.0, 51.8, 47.0, 40.3, 33.1, 29.8, 28.4, 23.0; LCMS $[M+H]^+$ 434.6, R_t = 3.6 min; HRMS calc. for $C_{22}H_{32}N_3O_4S$ MH^+: 434.2114. Found MH^+: 434.2089.

Tert-Butyl (S)-(6-amino-6-oxo-5-(((5-(pyridin-3-yl)thiophen-2-yl)methyl)amino)hexyl) carbamate ***[5]***

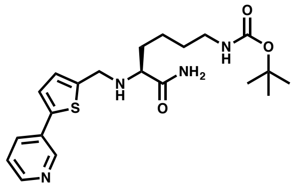

A solution of methyl N^6-(tert-butoxycarbonyl)-N^2-((5-(pyridin-3-yl)thiophen-2-yl)methyl)-L-lysinate (230 mg, 0.50 mmol) was made up in a 10 – 20 mL microwave vial (Biotage) with methanol (4 mL) before cooling to 0 ^{o}C whereupon magnesium nitride (270 mg, 2.70 mmol) was added in one portion. The vial was sealed and allowed to warm to room temperature in a water bath for 1 hr. The reaction was then heated to 80 ^{o}C for 24 hr, carefully monitoring the state of the cap over the first 3 hr. CAUTION: these reactions must be carried out behind a blast shield. The reaction was cooled to room temperature and vented carefully before diluting with chloroform (20 mL) and water (20 mL). The reaction was neutralised with HCl 3 N before the layers were separated and the aqueous layer further extracted with chloroform (3× 20 mL). The combined organics were dried over anhydrous magnesium sulphate, filtered and reduced *in vacuo*. The crude product was purified by column chromatography (Biotage, eluent 0 – 20% MeOH/DCM v/v) to give the *pyridine lysine amide* as a yellow solid (45 mg, 22% yield).

R_F (10% MeOH in DCM v/v) = 0.5. $\nu_{max.}$ 3327 cm^{-1} (N-H), 1670 cm^{-1} (C=O), 1167 cm^{-1} (C-O), 1026 cm^{-1} (C-N); ^{1}H NMR (400 MHz, $CDCl_3$) δ 8.77 (1H, dd, *J* 0.6, 1.9 Hz), 8.41 (1H, dd, *J* 1.2, 3.9 Hz), 8.01 (1H, ddd, *J* 1.3, 1.8, 6.4 Hz), 7.43 (1H, ddd, *J* 0.5, 3.9, 6.4 Hz), 7.35 (1H, d, *J* 2.9 Hz), 6.99 (1H, d, *J* 2.9 Hz), 3.99 (1H, d, *J* 11.9 Hz), 3.86 (1H, d, *J* 11.7 Hz), 3.19 (1H, t, *J* 5.3 Hz), 3.02 (2H, t, *J* 5.2 Hz), 1.72 – 1.57 (2H, m), 1.50 – 1.34 (13H, m); ^{13}C NMR (100 MHz, $CDCl_3$) δ 179.9, 158.5, 148.5, 146.7, 146.4, 140.0, 134.6, 132.7, 128.0, 125.7, 125.6, 79.8, 62.2, 41.1, 34.4,

30.8, 28.8, 24.1; LCMS $[M+H]^+$ 419.3, R_t = 1.2 min; HRMS calc. for $C_{21}H_{31}N_4O_3S$ MH^+: 419.2117. Found MH^+: 419.2130.

(S)-6-Amino-2-(((5-(pyridin-3-yl)thiophen-2-yl)methyl)amino)hexanamide **17**

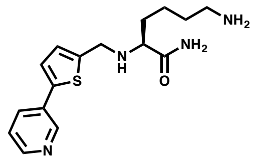

Tert-Butyl (*S*)-(6-amino-6-oxo-5-(((5-(pyridin-3-yl)thiophen-2-yl)methyl)amino)hexyl) carbamate (30 mg, 0.07 mmol) was dissolved in 4 M HCl in dioxane (4 mL) and stirred for 3 hr at room temperature. The reaction was reduced *in vacuo* to give the crude compound, which was left on the high vacuum line overnight and used without further purification. The *pyridine lysine amide* **17** isolated as the HCl salt, was a brown amorphous solid (30 mg, quantitative yield).

$\nu_{max.}$ 3379 cm^{-1} (N-H), 1630 cm^{-1} (C=O), 1033 cm^{-1} (C-N); ^1H NMR (400 MHz, CD_3OD) δ 9.28 (1H, s), 8.92 (1H, d, *J* 7.7 Hz), 8.83 (1H, d, *J* 5.2 Hz), 8.18 (1H, t, *J* 6.3 Hz), 7.82 (1H, d, *J* 2.8 Hz), 7.51 (1H, d, *J* 2.6 Hz), 4.58 (1H, d, *J* 14.2 Hz), 4.52 (1H, d, *J* 14.2 Hz), 4.08 (1H, t, *J* 5.7 Hz), 2.98 (2H, t, *J* 7.0 Hz), 2.12 – 1.96 (2H, m), 1.82 – 1.69 (2H, m), 1.63 – 1.52 (2H, m); ^{13}C NMR (100 MHz, CD_3OD) δ 170.8, 144.0, 141.1, 139.7, 139.5, 136.7, 135.3, 134.5, 129.5, 129.1, 60.5, 45.0, 40.2, 31.0, 28.0, 22.8; LCMS $[M+H]^+$ 319.3, R_t = 0.3 min; HRMS calc. for $C_{16}H_{23}N_4OS$ MH^+: 319.1593. Found MH^+: 319.1605.

Supplemental References

[1] A. Giesecke, M. Stewart, *Journal of Biological Chemistry* **2010**, *285*, 17628–17635.

[2] J. Sambrook, E. Fritsch, T. Maniatis, *Molecular Cloning: a Laboratory Manual*, Cold Spring Harbor Laboratory Press (Cold Spring Harbor Laboratory)., **1989**.

[3] D. Stock, O. Perisic, J. Löwe, *Prog. Biophys. Mol. Biol.,* **2005**, *88*, 311–327.

[4] K. Conde, M. Ankerson, U. Sensfuss, *Patent US2007185128A1*, **2007**.

[5] G. E. Veitch, K. L. Bridgwood, S. V. Ley, *Org Lett* **2008**, *10*, 3623–3625.

[6] N. D. Jabre, T. Respondek, S. A. Ulku, N. Korostelova, J. J. Kodanko, *J. Org. Chem.* **2010**, *75*, 650–659.

[7] K. Nakajima, F. Takai, T. Tanaka, K. Okawa, *Bulletin of the Chemical Society of Japan* **1978**, *51*, 1577–1578.

[8] N. Wang, J. Xiang, Z. Ma, J. Quan, J. Chen, Z. Yang, *J. Comb. Chem.* **2008**, *10*, 825–834.

[9] P. Watts, C. Wiles, S. J. Haswell, E. Pombo-Villar, *Tetrahedron,* **2002**, *58*, 5427–5439.

[10] J. E. Mangette, M. R. Johnson, V.-D. Le, R. A. Shenoy, H. Roark, M. Stier, T. Belliotti, T. Capiris, P. R. Guzzo, *Tetrahedron,* **2009**, *65*, 9536–9541.

[11] T. E. Barder, S. L. Buchwald, *Org Lett* **2004**, *6*, 2649–2652.
